# Economic, nutritional, and efficiency assessment of recycled casing soil and compost nutritional supplements for sustainable white button mushroom cultivation

**DOI:** 10.1371/journal.pone.0337146

**Published:** 2026-08-13

**Authors:** Ali Eslamizadeh, Sepideh Kalateh Jari, Ali Mohammadi Torkashvand, Mohammad Farsi, Mohamadreza Salehi Salmi

**Affiliations:** 1 Department of Agricultural Science and Engineering, SR.C., Islamic Azad University, Tehran, Iran; 2 Department of Biotechnology and Plant Breeding, Faculty of Agriculture, Ferdowsi University of Mashhad, Mashhad, Iran; 3 Department of Horticultural Science, Agricultural Sciences and Natural Resources University of Khuzestan, Ahwaz, Iran; Amity University Noida, INDIA

## Abstract

**Background:**

Optimizing white button mushroom (*Agaricus bisporus* L.) cultivation requires balancing economic, nutritional, and environmental goals—a challenge rarely addressed systematically. While recycled casing materials and nutritional supplements have been individually studied, their combined effects under multi-objective decision-making frameworks remain unexplored.

**Methods:**

This study introduces a two-phase framework to identify sustainable strategies based on producer priorities. In the first phase, a r.andomized split-plot field experiment (three replications, one year) evaluated seven casing soil formulations (including recycled materials and peat alternatives) and five nutritional supplements (soybean meal and corn grit at different levels). Yield, quality, and nutritional traits were assessed across three flushes. In the second phase, a goal programming (GP) model integrated these data to optimize three conflicting objectives: economic profit, a nutritional index (based on Mg, Zn, Cu, Mn, protein, and fiber), and the third-flush yield ratio.

**Results:**

The optimal combination was casing formulation C5 (20% Tima peat, 40% recycled soil, 25% fresh bagasse, 10% fermented bagasse, 5% calcium carbonate+perlite) with 1500 g/m^2^ corn grit (S5). This solution balanced all three objectives while incorporating 40% recycled material, supporting circular economy principles. Corn grit supplementation significantly enhanced yield (up to 36,669 g/m^2^) and economic profit, outperforming soybean meal. The GP-based framework demonstrated robust stability against weight variations (signal-to-noise ratio > 1.75), confirming its reliability for decision-making.

**Conclusions:**

The synergistic use of recycled casing soils with corn grit supplementation establishes a robust, agronomically sound model for commercial mushroom cultivation, delivering superior economic return while maintaining yield efficiency and nutritional quality. The validated GP framework offers growers a practical tool to systematically evaluate trade-offs and identify optimal cultivation strategies aligned with their specific priorities.

## 1. Introduction

Edible mushrooms provide substantial economic, nutritional, and ecological benefits. Their rapid growth, modest space requirements, and limited technical demands make them a sustainable source of income, particularly in rural communities [1,2,3,4]. Nutritionally, mushrooms are rich in protein, vitamins, minerals, and dietary fiber, helping to alleviate micronutrient deficiencies while offering a low-calorie, nutrient-dense food [5,6,7,8,9]. Environmentally, mushroom cultivation requires minimal land and water and can utilize agricultural residues, supporting circular economy principles through cost reduction and decreased impact [10,11]. Among cultivated species, the white button mushroom (*A. bisporus*) is the most widely produced worldwide [12,13]. Meeting its increasing demand requires improvements in both yield and quality. Production is highly dependent on two critical inputs: compost and casing soil [14]. Compost serves as the primary nutrient source, while casing soil plays a pivotal role in fruiting body initiation and development [15,16,17]. Compost quality directly influences yield and nutritional composition [18,15], and casing soil remains indispensable for successful mushroom formation [17].

The selection of Mg, Zn, Cu, and Mn as target micronutrients is based on their established roles in human nutrition and public health. Mg deficiency is linked to metabolic syndrome and hypertension; Zn deficiency affects ~17% of the global population, especially in cereal‑dependent regions [19]. Cu is essential for iron homeostasis and oxidative defense, while Mn acts as a cofactor for glycosyltransferases and Mn‑SOD [20]. Edible mushrooms, particularly Agaricus bisporus, effectively accumulate these minerals from the substrate without anti‑nutritional factors, making them suitable for mineral biofortification [21]. Thus, quantifying Mg, Zn, Cu, and Mn in fruiting bodies across different casing–supplement combinations allows us to identify agronomic practices that enhance micronutrient density, directly addressing hidden hunger—a dimension of food security often neglected in yield‑focused studies.

Compost composition decisively affects mushroom yield and nutritional quality. Strategic supplementation with nutrients, minerals, and beneficial microorganisms enhances productivity, improves quality, and shortens cultivation cycles [22,23,24,25,26,27]. Incorporating lignocellulosic materials such as bagasse strengthens sustainability by balancing nutrient availability and reducing reliance on conventional inputs. Compost enrichment also increases protein, vitamin, and bioactive compound content, delivering dual benefits in production efficiency and nutritional value [28,29,17]. Common supplements such as soybean meal (protein) and corn grit (carbohydrate) improve substrate nutrition, structural integrity, and microbial activity, enhancing both yield and quality [30,31,32,33]. However, the high cost of infrastructure, labor, fuel, and inputs—particularly protein-rich supplements like soybean meal—remains a major financial constraint, especially for small and medium-sized enterprises.

The mushroom industry has traditionally depended on peat-based casing soils, which remain standard due to their favorable technical and economic characteristics [34]. However, peat extraction poses significant environmental risks, including carbon emissions and ecosystem degradation [35,36]. In response, many countries have restricted peat extraction and promoted sustainable alternatives [37,38]. In this context, recycled casing soil from spent mushroom substrates and organic waste has emerged as a promising substitute. Research indicates that recycled casings support optimal mushroom growth and yield by supplying essential nutrients and maintaining adequate moisture. For example, incorporating recycled paper and composted agricultural residues can enhance product quantity and quality while reducing reliance on non-renewable resources. This approach also mitigates the environmental impacts of peat extraction and waste disposal [39,40,41,42]. Nevertheless, any alternative casing material must be assessed from two perspectives: first, its ability to match peat’s yield and quality; second, its economic viability for growers [43].

While the individual advantages of recycled casing soils and nutritional supplements are well documented [44], determining an optimal combination that concurrently satisfies competing economic and food security objectives remains a complex and unresolved issue. This complexity arises from intrinsic trade-offs between these goals; for example, a combination that maximizes yield may lack economic feasibility, whereas the most cost-effective alternative could diminish the nutritional quality of the final product. Addressing this conflict and identifying an optimal equilibrium within a multidimensional solution space necessitates a systematic, multi-objective approach capable of simultaneously modeling and optimizing profit, yield, and quality metrics. Such an approach constitutes the only scientifically robust pathway toward a sustainable and practical solution for the mushroom cultivation industry. To address this multi‑objective complexity, we employed goal programming (GP), a mathematical optimization technique suitable for problems with conflicting criteria [43,45–48]. The detailed formulation of our GP model, including constraints and weighting scenarios, is presented in Section 2.2.

This study introduces a novel multi-objective decision framework addressing a critical research gap in sustainable button mushroom cultivation. Previous studies have separately investigated recycled casing soils or nutritional supplements on yield and quality, but they have not systematically optimized their interactions under multi-objective settings. Existing literature often focuses on single objectives—maximizing yield or reducing costs—without balancing economic, nutritional, and efficiency goals in a unified framework. Moreover, integrating empirical agronomic data with multi-criteria models such as GP remains underexplored.

This study presents is an integrated two-phase experimental-modeling framework. First, rigorous field trials evaluate casing soil-supplement interactions across three harvest phases. Second, GP identifies optimal combinations that satisfy competing sustainability objectives based on producer priorities. The framework optimizes three criteria: economic profitability, nutritional quality, and production efficiency (minimizing low-yield phases). This approach bridges experimental agronomy and multi-criteria decision analysis, addressing the limitations of conventional single‑objective approaches. By integrating structured experimental data with mathematical optimization, our framework systematically identifies optimal combinations of recycled casing soils and nutritional supplements that balance economic viability with nutritional quality and operational efficiency. The ranking mechanism—minimizing deviations from weighted objective targets—provides a scientifically rigorous yet practical tool for growers to identify context-specific solutions aligned with their unique priorities (maximizing profit, enhancing nutritional value, or improving efficiency). This holistic approach resolves practical challenges in mushroom cultivation and offers a transferable paradigm for other agricultural sectors with multiple sustainability objectives. The study thus contributes to both the theory and practice of multi-criteria decision-making in sustainable agriculture.

The specific objectives are: (1) to evaluate seven casing soil formulations (including recycled materials) and five nutritional supplements on yield, quality, and nutritional composition of white button mushrooms through a replicated field experiment; (2) to develop a GP model integrating experimental data to optimize three conflicting objectives—economic profit maximization, nutritional quality enhancement, and production efficiency improvement (minimized third-flush yield share); and (3) to identify the most sustainable cultivation strategy via sensitivity analysis on objective weights, thereby providing an adaptable decision-support tool for growers under varying economic and environmental priorities.

## 2. Materials and methods

**Fig 1:**
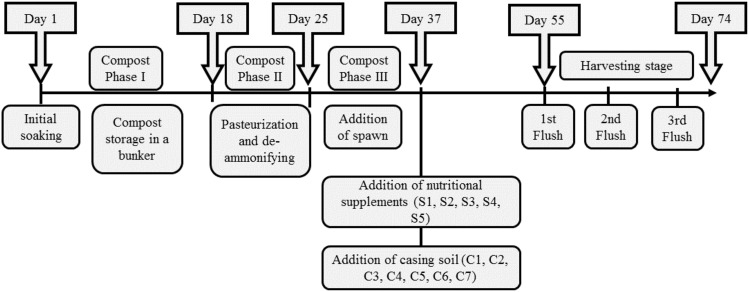
Overview of the experimental phase.

**Fig 2:**
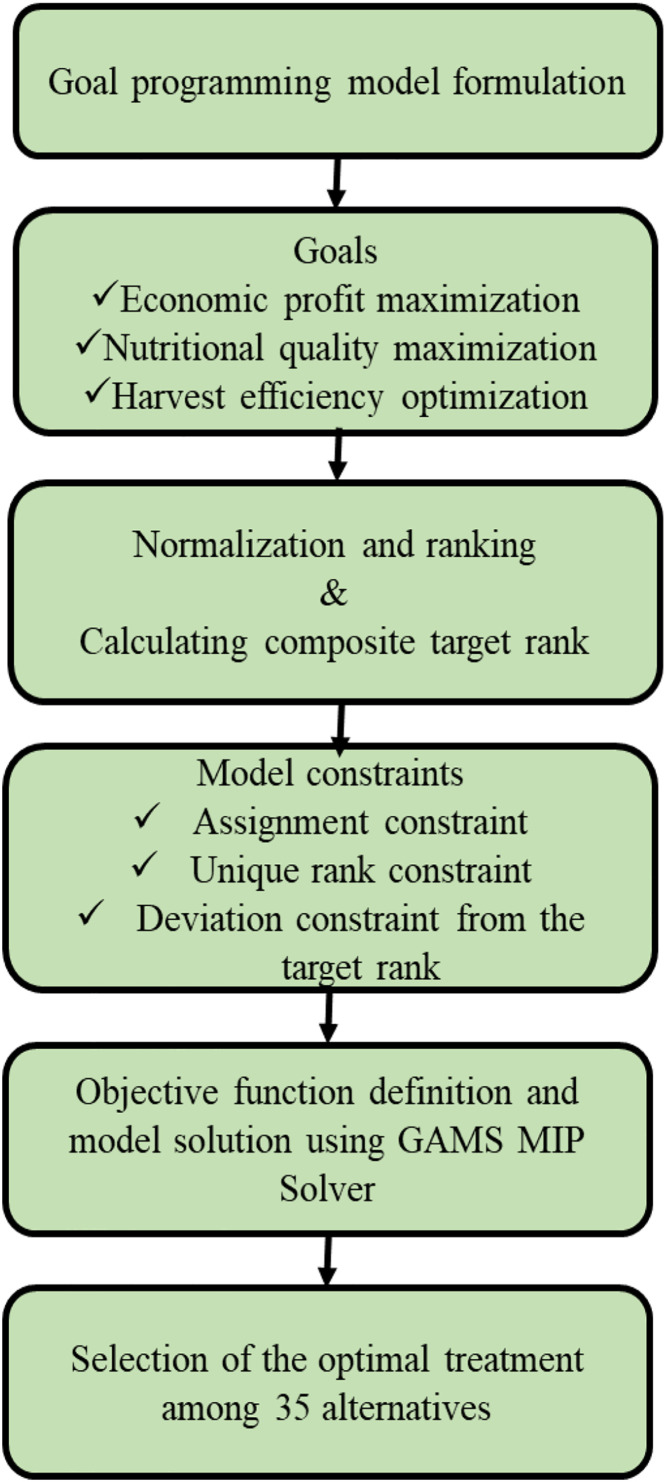
Overview of the modelling phase.

This study adopted a structured, two-phase approach to determine the optimal cultivation strategy for white button mushrooms. The process began with an extensive agronomic experiment designed to generate empirical data, which was later applied in a mathematical modeling phase focused on multi-objective optimization. As outlined in Section 2.1, the experimental phase employed a randomized split-plot design to assess the impact of seven casing soil formulations and five nutritional supplements on yield, quality, and nutritional composition (Fig 1). After measuring and collecting the yield data, analysis of variance of the indicators was performed. Subsequent mean comparisons were performed with the Tukey test at a 5% significance level. Statistical calculations and graph plotting were performed using Minitab and Excel software, respectively. Subsequently, Section 2.2 details the construction of a Goal Programming (GP) model that incorporated the experimental findings to identify the most effective treatment by concurrently optimizing three core objectives: economic profitability, nutritional enhancement, and harvest phase efficiency (Fig 2).

### 2.1. Experimental setup and procedures

#### 2.1.1. Study site and duration.

This study was conducted in 2024 in the white button mushroom cultivation halls of the Jolgah Dez Agricultural Company (Dezful Mushroom®), located in Dezful, Khuzestan Province, Iran (Latitude: 32.33925282130702, Longitude: 48.49924237273087).

#### 2.1.2. Experimental design.

The experiment was set up as a split-plot arrangement within a randomized complete block design (RCBD) with three replications. The main plots were assigned to seven different casing soil formulations, while the sub-plots consisted of five supplementation levels of soybean meal and corn grit added to the compost. The objective was to evaluate their effects on the yield, quality, and nutritional components of white button mushroom. The assumptions of normality and homogeneity of variances required for ANOVA were verified and satisfied across all variables, including percentage and ratio data; consequently, no data transformation was deemed necessary.

#### 2.1.3. Casing soil preparation.

Seven distinct casing soil formulations were prepared and evaluated to determine an optimal ratio for incorporating recycled agricultural wastes. This gradient design was applied to all treatments and was not based on a prior optimization process. Rather, the experimental approach allowed for a precise assessment of the incremental effects of increasing the proportion of recycled materials, aiming to identify the optimal balance between utilization efficiency and the physico-chemical properties of the casing layer compared to standard commercial soils. The formulations were as follows:

C1: 95% Imported BVB® (Netherlands) casing soil mixed with 5% calcium carbonate (2.5%) and perlite (2.5%).

C2: 95% Imported Tima peat® (Armenia) casing soil mixed with 5% calcium carbonate (2.5%) and perlite (2.5%).

C3: A mixture of 20% BVB, 40% recycled spent casing soil, 25% fresh bagasse, 10% fermented bagasse, and 5% calcium carbonate (2.5%) with perlite (2.5%).

C4: A mixture of 10% BVB, 50% recycled spent casing soil, 25% fresh bagasse, 10% fermented bagasse, and 5% calcium carbonate (2.5%) with perlite (2.5%).

C5: A mixture of 20% Tima peat, 40% recycled spent casing soil, 25% fresh bagasse, 10% fermented bagasse, and 5% calcium carbonate (2.5%) with perlite (2.5%).

C6: A mixture of 10% Tima peat, 50% recycled spent casing soil, 25% fresh bagasse, 10% fermented bagasse, and 5% calcium carbonate (2.5%) with perlite (2.5%).

C7: A mixture of 60% recycled spent casing soil, 25% fresh bagasse, 10% fermented bagasse, and 5% calcium carbonate (2.5%) with perlite (2.5%).

#### 2.1.4. Recycled casing soil preparation.

Spent casing soil from previous cultivation cycles was collected into piles. Primary leaching was conducted for one week using a sprinkler system. The material was then analyzed for key parameters, including electrical conductivity (EC), pH, organic carbon, total nitrogen content, organic matter, and carbon-to-nitrogen ratio, according to the Iranian National Standards (No. 1371). The leaching process was repeated 4–6 times until the optimal EC range of 800–1500 µS/cm was achieved. This specific threshold was selected based on established mushroom cultivation standards [49]. The upper limit of 1500 µS/cm provides a safe margin below the approximate inhibitory level of 2000 µS/cm, where higher EC can cause osmotic stress and inhibit mycelial growth, while the lower limit of 800 µS/cm ensures sufficient nutrient availability. The entire process of producing the recycled casing soil took between 4–6 weeks.

#### 2.1.5. Bagasse fermentation process.

Fresh bagasse was piled and initially leached for one week. Subsequently, the piles were turned and aerated monthly using a loader while being kept saturated with water. This fermentation and turning process was repeated over six months in approximately six cycles. The six-month period was determined through pre-commercial trials, with the primary goal of reducing the high concentration of residual molasses (soluble carbohydrates) and lowering the electrical conductivity (EC) to a level suitable for use as a cultivation substrate. This extended timeframe allowed for the gradual and effective reduction of soluble sugars and salts, ultimately bringing the EC of the bagasse below the target threshold of 1500 µS/cm, which is considered acceptable for the intended application. After this period, the bagasse was considered fully fermented and ready for use in the casing soil mixtures.

#### 2.1.6. Compost Preparation.

Phase III compost was used in this study. The compost was produced through a standard three-phase process:

Phase I: Raw materials, primarily wheat straw, chicken manure, and gypsum, were mixed, moistened, and homogenized. Due to intense microbial activity, the compost temperature reached 80–85°C. This phase lasted for two weeks.

Phase II: This phase consisted of pasteurization and conditioning. The compost was first pasteurized at 57–60°C for 12 hours. The temperature was then reduced to 45–50°C for three days to complete the ammonia removal process. Phase II lasted approximately 6–7 days.

Phase III: After Phase II, the compost was cooled to 25°C. Upon removal from the tunnels, it was inoculated with spawn (*A bisporus* L., Strain 737). The spawned compost was then transferred to another tunnel for mycelial colonization. An ideal temperature of 25°C was maintained for 14–16 days to complete vegetative mycelial growth, resulting in finished Phase III compost, which was then transferred to the cultivation halls for the experiment.

The following supplements were added to the Phase III compost:

S1: Control (no supplement).

S2: Soybean meal at 1000 g/m².

S3: Soybean meal at 1500 g/m².

S4: Corn grit at 1000 g/m².

S5: Corn grit at 1500 g/m².

These specific application rates were selected based on the common commercial range for nitrogen-rich supplements (typically 750–1500 g/m^2^) to evaluate the effect of two effective and practically relevant levels on compost efficiency and mushroom yield.

#### 2.1.7. Cultivation conditions.

Experimental units (replicates) were established by marking specific areas (1 m × 1.4 m) on the cultivation shelves. After filling the halls with Phase III compost, the nutritional supplements were applied according to the treatments. The respective casing soils were then applied onto the compost surface. The beds were maintained at 25°C for five days to allow mycelial colonization into the casing layer (spawn run).

To stimulate further mycelial growth, ruffling (casing soil mixing) was performed, which lasted for three hours. Two days after ruffling, aeration was initiated for four days to induce pinhead formation (initiation of fruiting bodies). The mushroom fruiting bodies were then allowed to develop over an eight-day growth period.

Throughout the cultivation cycle, environmental conditions in the growing hall, including temperature, humidity, and CO₂ levels, were automatically controlled and monitored using an automation system. Irrigation of the experimental units was managed via a sprinkler system, and all environmental parameters were maintained according to the standard operating procedures of the Jolgah Dez Agricultural Company.

#### 2.1.8. Harvesting procedure.

The mushrooms were harvested in three distinct flushes. Harvesting commenced when the mushrooms reached maturity, characterized by a fully formed cap before veil break. The mushrooms were carefully picked by twisting and cutting at the base using a sterilized sharp knife to prevent damage to the surrounding mycelium and developing pins. The first flush was harvested over a period of 5–6 days. After a two-day interval, the second flush was collected over another 5–6 days. Following another two-day rest period, the third and final flush was harvested over a further 5–6 days. The total cultivation cycle, from spawning to the end of the third flush, was completed within 31–33 days.

#### 2.1.9. Yield assessment.

Following each daily harvest, the total yield from each experimental unit was immediately measured using a digital balance with a precision of 1 g. daily yields were aggregated to determine the total yield for each flush. The cumulative yield across all three flushes was then calculated for each experimental unit. The contribution of each flush to the total yield was expressed as a percentage, using the formula provided by Hammond and Nichols [50]:


$$\begin{array}{c} The\;share\;of\;each\;flash\;in\;the\;total\;yield\;(\%)\;=\;(Flush\;Yield\;/\;Total\;Yield)\;\times \;\text{1}\text{0}\text{0} \end{array}$$ (1)

After each harvest, mushrooms were sorted based on commercial quality. Mushrooms with open veils, misshapen form, or undersized appearance—deemed unappealing to consumers—were classified as second-grade. These were separated and weighed. The proportion of second-grade mushrooms was determined for each experimental unit using the following formula:


$$\begin{array}{c} The\;proportion\;of\;second-grade\;mushrooms\;(\%)\;=\;(Weight\;of\;Second-grade\;Mushrooms\;/\;Total\;Weight)\;\times \;\text{1}\text{0}\text{0} \end{array}$$ (2)

#### 2.1.10. Analysis of nutritional properties and mineral content.

For the analysis, three independent replicates (n = 3) were prepared. Each replicate was a composite sample derived from four similarly-sized fruiting bodies, which were dried, powdered, and homogenized together. The nutritional composition of the mushroom fruiting bodies was analyzed as follows:

**Crude protein:** The crude protein content was determined based on the total nitrogen content, measured using the standard Kjeldahl method involving digestion, distillation, and titration. This primary volumetric method calculates nitrogen content directly from the titer and volume of the standard sulfuric acid consumed, eliminating the need for a calibration curve. Method reliability was ensured by analyzing all samples in triplicate, running method blanks in each batch, and validating accuracy via a recovery test using Glycine as a certified reference material, which yielded a recovery rate of 98–102%. The nitrogen content was calculated using Eq. 3:


$$\begin{array}{c} Nitrogen\;(\%)\;=\;(Volume\;of\;acid\;\times \;Normality\;of\;acid\;\times \;\text{1}.\text{4})\;/\;Sample\;Weight \end{array}$$ (3)

Subsequently, the crude protein percentage was calculated by multiplying the nitrogen percentage by the specific conversion factor of 4.38 (Eq. 4). This factor, instead of the general factor of 6.25, was applied as it is recommended for mushrooms due to their distinct non-protein nitrogen composition and amino acid profile, in accordance with international food composition standards [51].


$$\begin{array}{c} Crude\;Protein\;(\%)\;=\;Nitrogen\;(\%)\;\times \;\text{4}.\text{3}\text{8} \end{array}$$ (4)

Crude fiber: The crude fiber content was determined in triplicate (n= = 3) according to the standard gravimetric procedure, with the following specific details. Briefly, 2 g of dried and powdered mushroom sample was subjected to sequential digestion under reflux: first with 150 mL of 0.255 N sulfuric acid, followed by 150 mL of 0.313 N sodium hydroxide solution. After each digestion step, the mixture was filtered through a Whatman No. 1 filter paper, and the residue was thoroughly washed with distilled water. The filter paper containing the residue was then dried to a constant weight in an oven at 70°C for 48 hours, weighed, and subsequently ashed in a muffle furnace at 600°C for 4 hours. The crude fiber content was calculated based on the weight loss upon ashing, with correction for the ash content of the filter paper. The results are presented as the mean  ±  standard deviation of the three independent replicates.

**Mineral content:** The concentrations of magnesium (Mg), copper (Cu), zinc (Zn), and manganese (Mn) were determined using Inductively Coupled Plasma Optical Emission Spectrometry (ICP-OES; Model Optima 8300, PerkinElmer). The analytical performance parameters of the instrument for each element are summarized in Table 1. Method accuracy was confirmed via a recovery test using spiked samples, yielding recoveries of 94–104% for all elements. Prior to analysis, 2 g of the dried and powdered mushroom sample was dry-ashed in an electric furnace at 550°C, and the ash was dissolved in acid [52]. The results are expressed in parts per million (ppm) on a dry weight basis.

**Table 1:** ICP-OES analytical parameters: linear range, limit of detection (LOD), and limit of quantification (LOQ).

Element	Linear Range (µg/L)	LOD (µg/L)	LOQ (µg/L)
Mg	10–5000	0.5	1.7
Mn	2–2000	0.2	0.7
Zn	5–3000	0.4	1.3
Cu	3–2500	0.3	1.0

### 2.2. Mathematical modeling framework

Drawing on empirical data from field experiments, a goal programming (GP) model was constructed to determine the most effective cultivation strategy. This model aimed to identify the optimal treatment by concurrently optimizing three primary objectives within the framework of existing constraints. The coding of the treatments (including five types of nutritional supplements and seven casing soil formulations) for constructing the GP model is summarized in Table 2.

**Table 2:** Coding of the experimental treatments.

	S1	S2	S3	S4	S5
C1	a1	a2	a3	a4	a5
C2	a6	a7	a8	a9	a10
C3	a11	a12	a13	a14	a15
C4	a16	a17	a18	a19	a20
C5	a21	a22	a23	a24	a25
C6	a26	a27	a28	a29	a30
C7	a31	a32	a33	a34	a35

#### 2.2.1. Model objectives.

**2.2.1.1. Economic profit maximization (*π*):** The total profit per square meter (π) is calculated as the total revenue from the sale of first-grade and second-grade mushrooms minus the sum of all production costs, expressed by the following formula:


$$\begin{array}{c} \text{Max }\pi_{a}=\left(P\text{1}*{Y\text{1}}_{a}\right)+\left(P\text{2}*{Y\text{2}}_{a}\right)-({C\text{1}}_{a}+{C\text{2}}_{a}+CLG+CLH+CG+CE+CW) \end{array}$$ (5)

Where a denote the treatments (alternatives). *P1* and *Y1* represent the price (USD per kg) and yield of first-grade mushrooms (kg per square meter), *P2* and *Y2* represent the price (USD per kg) and yield of second-grade mushrooms (kg per square meter), *C1* denotes the cost of the casing soil preparation (USD kg per square meter), *C2* the cost of dietary supplements (USD per square meter), *CLG* the labor cost for the growth phase (USD per square meter), *CLH* the labor cost for harvesting (USD per square meter), and CG, CE, and CW represent the costs of gas, electricity, and water (USD per square meter), respectively.

**2.2.1.2. Nutritional quality maximization (*FEI*):** This goal sought to favor alternatives that produced mushrooms with the highest aggregated nutritional value (Eq. 6). A composite food elements index (FEI) was calculated as the normalized (NV) sum of key minerals (Mg, Mn, Zn, and Cu) and macronutrients (Crude Protein and Crude Fiber), where normalization was performed using the Eq. (7):


$$\begin{array}{c} {Max\;FEI}_{a}={NV\;of\;Mg}_{a}+{NV\;of\;Mn}_{a}+{NV\;of\;Zn}_{a}+{NV\;of\;Cu}_{a}+{NV\;of\;Crude\;Protein}_{a}+{NV\;of\;Crude\;Fiber}_{a} \end{array}$$ (6)

$$\begin{array}{c} NV\;of\;Elements=\frac{Data-Minimum}{Maximum-Minimum} \end{array}$$ (7)

**2.2.1.3. Minimizing the ratio of the third flush yield (*RY*):** This goal intended to minimize the unproductive phase of the cultivation cycle by prioritizing alternatives with a lower ratio of the third flush yield to the total yield. A lower ratio indicates a more concentrated and efficient harvest in the earlier, more productive flushes.

#### 2.2.2. Normalization and ranking.

To ensure comparability among the disparate objectives—economic profit (π), nutritional index (FEI), and third-flush yield ratio (RY)—which are expressed in different scales and units of measurement, a preference ranking normalization method was employed. This approach facilitates the integration of incommensurable objectives in multi-criteria decision-making problems with discrete alternatives (reference to MCDM literature). Accordingly, each alternative (a) was assigned a rank for each goal. The ranking was performed as follows:

- For the profit (*π*) and nutritional quality (*FEI*) objectives, alternatives were ranked in descending order (higher value = better rank = lower rank number).
- For the harvest ratio (*RY*) objective, alternatives were ranked in ascending order (lower ratio = better rank = lower rank number).

These ranks are denoted as Rank_pr(a), Rank_fe(a), and Rank_ry(a), respectively.

Subsequently, a composite target rank (Target_Rank) for each alternative was calculated as the weighted sum of its single-objective ranks (Eq. 8):


$$\begin{array}{c} {Target\_Rank}_{a}=\left(w_{\text{1}}*{Rank\_pr}_{a}\right)+{(w}_{\text{2}}*{Rank\_fe}_{a})+(w_{\text{3}}*{Rank\_ry}_{a}) \end{array}$$ (8)

In this equation, the coefficients w1, w2, and w3 reflect the relative importance assigned to the economic, nutritional, and efficiency objectives by the decision-maker. The values of these weights were defined to represent different prioritization scenarios. The selection of specific weight values (e.g., 2/3, 1/6, 1/6) was based on a preliminary sensitivity analysis. This analysis demonstrated that these weight combinations led to significant and interpretable differences in the final ranking of alternatives. Therefore, they effectively model distinct decision-maker priorities (e.g., strong emphasis on a single objective versus a balanced approach) and provide actionable insights for growers with varied goals.

#### 2.2.3. Model constraints.

The decision variable in the current model is denoted by $h_{a,r}$ (binary variable) which represents the assigned rank to 35 alternatives based on the simultaneous fulfillment of the objectives. The GP model was subject to the following set of constraints to ensure a feasible and logical solution:

**Assignment constraint:** Each alternative must be assigned to exactly one rank position (r).


$$\begin{array}{c} \sum_{r}h_{a,r}=\text{1}\;\forall \;a \end{array}$$ (9)

**Unique rank constraint:** Each rank position (r) must be assigned to exactly one alternative. This ensures a strict, non-tied ranking from best (rank 1) to worst (rank 35).


$$\begin{array}{c} \sum_{a}h_{a,r}=\text{1}\;\forall \;r \end{array}$$ (10)

**Target reach constraint:** This primary constraint captures the deviation between the assigned rank and the target rank for each alternative. The rank allocated to an alternative must equal the sum of Target_Rank and the associated deviation variables including dev_plus and dev_minus which represent the surplus and shortfall relative to the target, respectively (Eq. 11).


$$\begin{array}{c} \sum_{r}h_{a,r}\;-\;{dev\_plus}_{a}+{\;dev\_minus}_{a}=\;{Target\_Rank}_{a}\;\forall a \end{array}$$ (11)

#### 2.2.4. Model ultimate goal.

The ultimate objective of the GP model was to minimize the total absolute deviation from the target ranks across all alternatives. This approach identifies a ranking that approximates the ideal weighted ranking according to the preferences of the decision-maker.


$$\begin{array}{c} Min\;Z=\sum_{a}{{dev\_plus}_{a}+dev\_minus}_{a} \end{array}$$ (12)

#### 2.2.5. Solution method.

The formulated model is a Mixed-Integer Programming (MIP) model due to the binary variables $h_{a}$. The model was implemented in the General Algebraic Modeling System (GAMS) and solved using a standard MIP solver to global optimality, thereby identifying the single best ranking of the 35 cultivation alternatives that minimizes the total deviation from the stated goal (S1 Text).

## 3. Results

The findings of this study are presented in two main sections. The first section examines the yield of button mushrooms as influenced by casing soil type, nutritional supplements in the compost substrate, and their interaction. The second section details the calculation of economic profit, nutritional characteristics, and the third-flush to total yield ratio to determine the optimal casing soil and nutritional supplement combination from production efficiency, economic, and nutritional perspectives.

### 3.1. Yield Analysis

Analysis of variance (Table 3) showed that casing soil type, nutritional supplement type, and their interaction each significantly affected total yield (p < 0.05), confirming that both factors—and their combination—determine overall productivity. S1 Table provides complete ANOVA tables for all nutritional components (protein, fiber, Cu, Mg, Mn).

**Table 3:** Analysis of variance of the effect of casing soil type, compost supplements and their interaction on total yield.

Source	df (degree freedom)	Total yield	p-value
Block	2	911781^**^	0.003
Casing soil (a)	6	16991108 ^**^	0.001
Error a	12	11868	0.950
Supplement (b)	4	417438673 ^**^	0.000
(a*b)	24	1022538^**^	0.001
Error b Total	56 104	16910 436392878	
CV (%)		11.45	

*, **, and ns indicate significance at the 5% level, 1% level, and non-significance, respectively.

*Note*: Each treatment combination was replicated three times (n = 3 per treatment)

**Fig 3:**
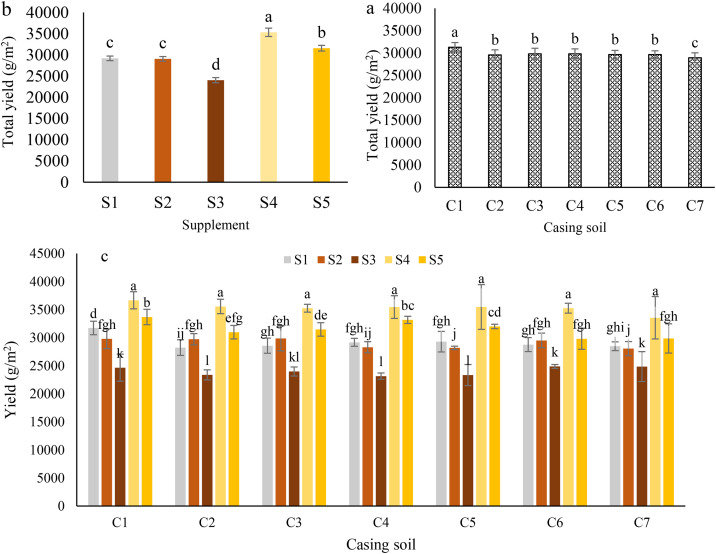
Effect of casing soil and nutritional supplements on total yield of button mushroom. *Note*: In each graph, similar letters indicate no significant difference between treatments at the 5% probability level of Tukey’s test, Error bars represent the standard error of the mean (±SE) calculated from the raw data. Each treatment combination was replicated three times (n = 3 per treatment).

Among casing soils, C1 (95% imported BVB peat with 5% calcium carbonate and perlite) produced the highest total yield with 31,307 g/m^2^, while C7 (60% recycled soil, no peat) produced the lowest with 28,959 g/m^2^ (Fig 3a). Notably, the yield difference between the best and worst casings was only about 8%, suggesting that casing type alone is not the dominant driver of yield. In contrast, supplement type had a much stronger effect. Corn grit at 1000 g/m^2^ (S4) gave the highest yield (35,318 g/m^2^), whereas soybean meal at 1500 g/m^2^ (S3) gave the lowest (24,030 g/m^2^) (Fig 3b). This 47% difference indicates that supplement selection is more critical than casing choice for maximizing yield.

The highest absolute yield (36,669 g/m^2^) occurred with C1 (BVB peat) combined with S4 (corn grit 1000 g/m^2^). However, this was not statistically different from S4 combined with C2 through C6, meaning that corn grit at 1000 g/m^2^ consistently elevated yield across most casing types (Fig 3c). Conversely, the lowest yield (23,148 g/m^2^) occurred with C4 (10% BVB, 50% recycled) plus S3 (soybean meal 1500 g/m^2^), but similarly low yields were observed when S3 was combined with C2, C3, C4, or C5. This pattern implies that high-dose soybean meal suppresses yield regardless of casing quality.

### 3.2. Multi-objective analysis

#### 3.2.1. Economic profit analysis.

**Fig 4:**
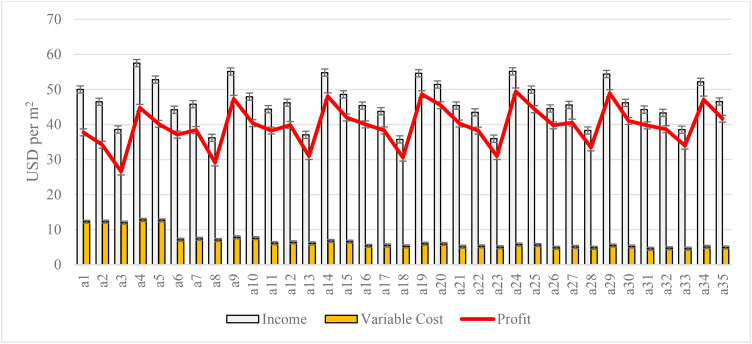
Revenue, variable cost, and economic profit of button mushrooms for different alternatives. *Note*: Error bars represent the standard error of the mean (±SE) calculated from the raw data. Each treatment combination was replicated three times (n = 3 per treatment).

In the present study, the economic profit objective was evaluated as one of the most important decision-making criteria for edible mushroom producers to select the best treatment (alternative). Consequently, the revenue from edible mushroom production, variable production costs, and the economic profit for different alternatives were calculated and are presented in Fig 4.

The highest revenue alternatives (a4, a24, a9, a14, a19, a29) all shared the same supplement: S4 (corn grit 1000 g/m^2^). Casing type varied among these top earners (ranging from C1 to C6), indicating that supplement choice determines revenue more than casing formulation. The only exception was a34 (C7 with S4), which yielded lower revenue than a5 (C1 with S5, corn grit 1500 g/m^2^), suggesting that extremely recycled casings like C7 may attenuate even a good supplement’s revenue potential.

Variable costs were most influenced by casing type. The highest costs were associated with C1 (imported BVB peat), seen in alternatives a4, a5, a2, a1, and a3. Conversely, the lowest costs occurred in alternatives with high proportions of recycled soil (50% or more, e.g., a31, a33, a32, a28, a26). This confirms that substituting recycled casing material directly reduces production costs.

The highest net profits were achieved by a24 (C5 + S4: 49.40 USD per square meter), a29 (C6 + S4: 48.92), a19 (C4 + S4: 48.63), a14 (C3 + S4: 48.03), a9 (C2 + S4: 47.31), and a34 (C7 + S4: 47.08). Notably, a24 (C5: 20% Tima peat, 40% recycled) outperformed even the all-peat C1 combinations, demonstrating that profitability does not require expensive imported casing—rather, it requires a cost-effective casing paired with an effective supplement. The lowest profits (a3, a8, a18, a23, a13) all used soybean meal at 1500 g/m^2^ (S3), confirming that high-dose protein supplementation is economically detrimental regardless of casing type.

#### 3.2.2. Nutritional characteristics analysis.

**Fig 5:**
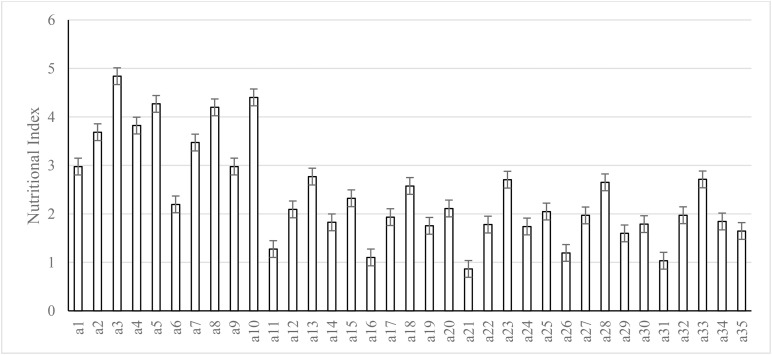
Nutritional index of button mushrooms for different alternatives. *Note*: Error bars represent the standard error of the mean (±SE) calculated from the raw data. Each treatment combination was replicated three times (n = 3 per treatment).

To evaluate nutritional quality, a Food Elements Index (FEI) was constructed based on the concentrations of four dietarily essential minerals (Mg, Zn, Cu, Mn) and two macronutrients (crude protein, crude fiber), all measured on a dry weight basis (S2 Table). These minerals were selected for their established roles in human metabolic health, including enzymatic cofactor functions, antioxidant defense, and metabolic homeostasis. Normalized values were summed to generate a dimensionless FEI (Fig 5). The results indicated that the five alternatives with the highest nutritional index values were, in order, alternatives a3, a10, a5, a8, and a4, with approximate values of 4.84, 4.40, 4.27, 4.20, and 3.82, respectively. Notably, four of these five alternatives received the highest soybean meal supplementation (S3: 1500 g/m^2^), while a10 received corn grit at 1500 g/m^2^ (S5). In contrast, the five alternatives with the lowest nutritional index values were a21, a31, a16, a26, and a11, with approximate values of 0.86, 1.03, 1.10, 1.19, and 1.27, respectively. All low‑index alternatives received no nutritional supplement (S1), regardless of casing type. A clear dose–response pattern emerged: increasing soybean meal from 1000 g/m^2^ (S2) to 1500 g/m^2^ (S3) consistently raised FEI across all casing soils, with a substantial average increase. For corn grit, the increase from 1000 g/m^2^ (S4) to 1500 g/m^2^ (S5) was marginal, suggesting that mineral uptake approaches saturation at the lower dose (1000 g/m^2^). These patterns indicate that nitrogen‑rich soybean meal enhances mineral accumulation more effectively than carbohydrate‑rich corn grit, albeit at the cost of reduced total yield (Fig 3c). Therefore, the best nutritional characteristics were achieved when using a casing soil composed of 95% imported BVB from the Netherlands and 5% calcium carbonate with perlite, combined with a compost nutritional supplement of 1500 g/m^2^ soybean meal (alternative a3). Conversely, the lowest nutritional characteristics index was observed when using the C5 casing soil formulation (20% Tima peat, 40% recycled soil, 25% fresh bagasse, 10% fermented bagasse, and 5% calcium carbonate with perlite) and no nutritional supplement in the compost (alternative a21). This inverse relationship between yield and nutritional density is further analyzed in Section 4.

#### 3.2.3. Third flush yield ratio analysis.

**Fig 6:**
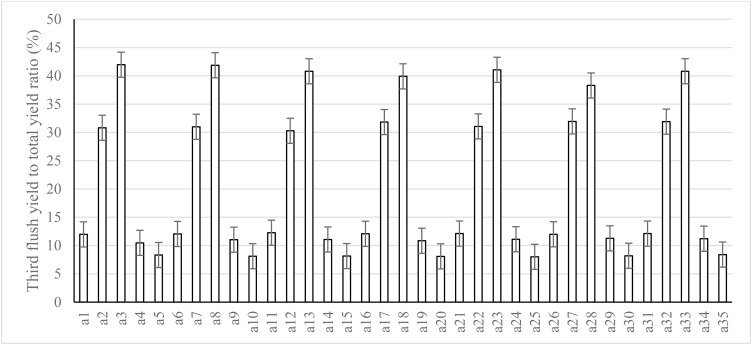
Ratio of the third flush yield to the total yield of button mushrooms for different alternatives. *Note*: Error bars represent the standard error of the mean (±SE) calculated from the raw data. Each treatment combination was replicated three times (n = 3 per treatment).

The third criterion—production efficiency—was quantified as the ratio of third-flush yield to total yield. A lower ratio indicates that most harvest occurs in the first two flushes, allowing growers to terminate the cycle earlier, saving energy and labor. Fig 6 shows this ratio across all alternatives.

The seven lowest ratios (a25: 8.01%, a20: 8.09%, a10: 8.12%, a15: 8.14%, a30: 8.19%, a5: 8.33%, a35: 8.41%) all used S5 (corn grit 1500 g/m^2^) as the supplement, regardless of casing type (casing ranged from C1 to C7). This consistency indicates that supplement choice—not casing—determines flush distribution. Corn grit at the higher application rate was able to concentrate approximately 92% of total yield in the first two flushes.

The highest ratios (a3: 41.96%, a8: 41.87%, a23: 41.06%, a33: 40.81%, a13: 40.80%, a18: 39.91%, a28: 38.29%) all used S3 (soybean meal 1500 g/m^2^). This means that with S3, nearly 40% of total yield comes from the third flush, making early termination uneconomical. For growers prioritizing cycle time and energy savings, S5 is therefore the preferred supplement, while S3 should be avoided because it forces a longer production cycle.

#### 3.2.4. Multi-objective analysis.

**Fig 7:**
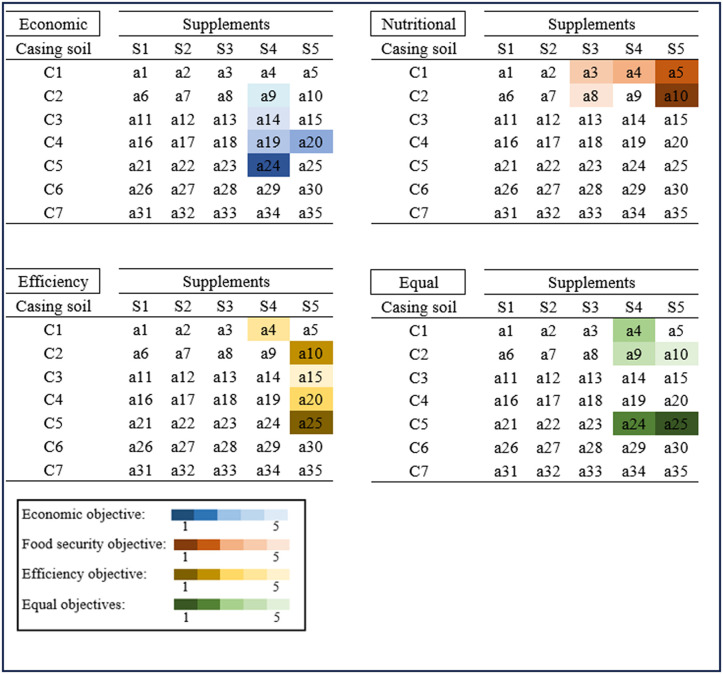
Sensitivity analysis to evaluate the influence of different casing soils and compost nutritional supplements on the ranking of alternatives.

The goal programming model considered three objectives simultaneously: economic profit maximization, nutritional index maximization, and third-flush yield ratio minimization. The model ranked all 35 alternatives under four weight scenarios (economic priority, nutritional priority, efficiency priority, and equal weights). Table 4 and Fig 7 present the results. To validate the model and assess the robustness of its recommendations, a comprehensive sensitivity analysis was performed by systematically varying the weights assigned to each objective. This analysis serves a dual purpose: it demonstrates how optimal decisions shift with changing decision-maker priorities, and it confirms the stability and logical consistency of the model.

**Table 4:** Ranking of alternatives for simultaneous multi-objective achievement under varying priority weights.

Multi-objectives	Economic	Food security	Efficiency	Ranking
Economic	2/3	1/6	1/6	a24 > a20 > a19 > a14 > a9 > … > a13 > a33 > a23 > a8 > a3
Nutritional characteristics	1/6	2/3	1/6	a10 > a5 > a4 > a3 > a8 > … > a21 > a31 > a16 > a26 > a11
Efficiency	1/6	1/6	2/3	a25 > a10 > a20 > a4 > a15 > … > a13 > a18 > a23 > a8 > a3
Equal objectives	1/3	1/3	1/3	a25 > a24 > a4 > a9 > a10 > … > a11 > a3 > a8 > a31 > a21

When economic profit was prioritized (weights of 2/3 for profit, 1/6 for nutrition, 1/6 for efficiency), alternatives a24, a20, a19, a14, and a9 ranked highest, while a13, a33, a23, a8, and a3 ranked lowest. The best alternative under this scenario (a24) combined C5 casing with S4 supplement (corn grit 1000 g/m^2^). Under this weighting, the choice of supplement influenced producer decisions more than casing type.

When nutritional quality was prioritized (1/6 for profit, 2/3 for nutrition, 1/6 for efficiency), the rankings shifted dramatically. Alternatives a10, a5, a4, a3, and a8 became the best, while a21, a31, a16, a26, and a11 were the worst. Here, casing type had greater influence than supplement, with imported peat-based casings C2 and C1 performing best. Combining these casings with soybean meal at 1500 g/m^2^ or corn grit at 1500 g/m^2^ effectively maximized nutritional characteristics.

When production efficiency (minimizing third-flush ratio) was prioritized (1/6 for profit, 1/6 for nutrition, 2/3 for efficiency), the best alternatives were a25, a10, a20, a4, and a15, while the worst were a13, a18, a23, a8, and a3. Under this scenario, supplement type again played a more critical role than casing type. Corn grit at 1500 g/m^2^ (S5) combined with C3, C4, C2, or especially C5 casings effectively minimized the third-flush yield share, allowing for potential elimination of the third flush with associated energy and cost savings.

When equal importance weights were assigned to all three objectives (1/3 each), the top alternatives were a25, a24, a4, a9, and a10. The best overall was a25 (C5 with S5, corn grit 1500 g/m^2^), followed closely by a24 (C5 with S4, corn grit 1000 g/m^2^). This indicates that the C5 casing (20% Tima peat, 40% recycled soil, 25% fresh bagasse, 10% fermented bagasse, and 5% calcium carbonate with perlite) paired with corn grit (either 1000 or 1500 g/m^2^) provides the most balanced solution across economic, nutritional, and efficiency goals. Importantly, C5 contains 40% recycled material, demonstrating that sustainability through peat reduction does not conflict with multi-objective performance but is rather integral to the optimal solution.

### 3.3. Model validation and robustness check

To rigorously assess the robustness and validity of the proposed multi-objective ranking model against potential uncertainties in decision-makers’ preferences, a Monte Carlo simulation study was conducted. While predetermined weights were initially established to rank alternatives based on higher priority assigned to each objective using the goal programming method, it is well-known that such weights may be subject to minor perturbations due to judgmental inconsistencies or shifting priorities. Accordingly, 100 random weight vectors were generated for each priority scenario (economic profit, food security, and efficiency) under two main constraints:

1. The sum of the weights equals 1.
2. The weight of the prioritized objective was maintained as the highest among the three criteria.

**Fig 8:**
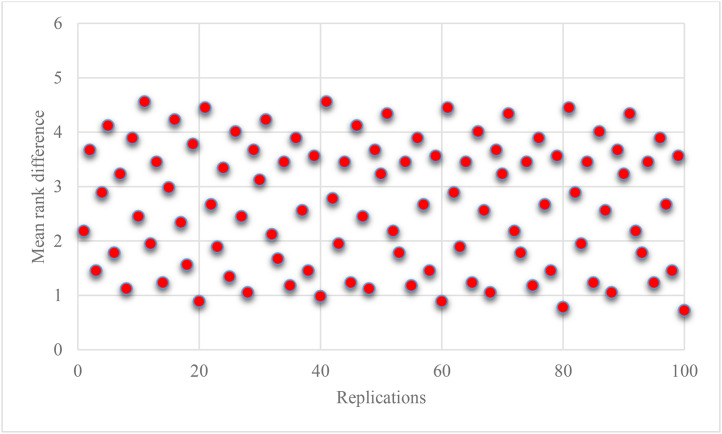
Scatter plot of mean rank difference of simulated alternatives under perturbed objective weights with higher priority to economic (100 Monte Carlo replications).

**Fig 9:**
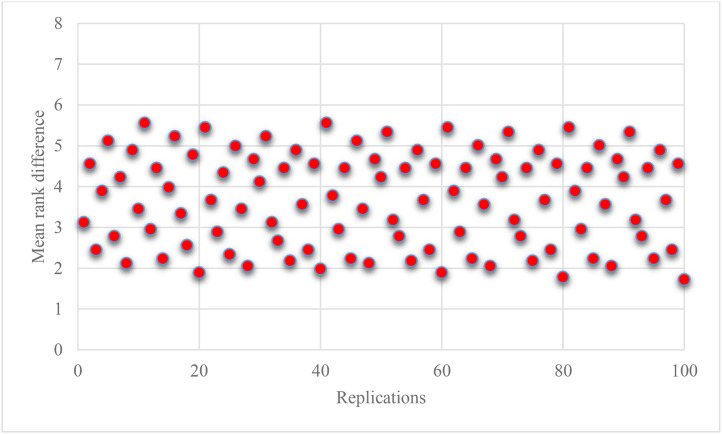
Scatter plot of mean rank difference of simulated alternatives under perturbed objective weights with higher priority to food security (100 Monte Carlo replications).

**Fig 10:**
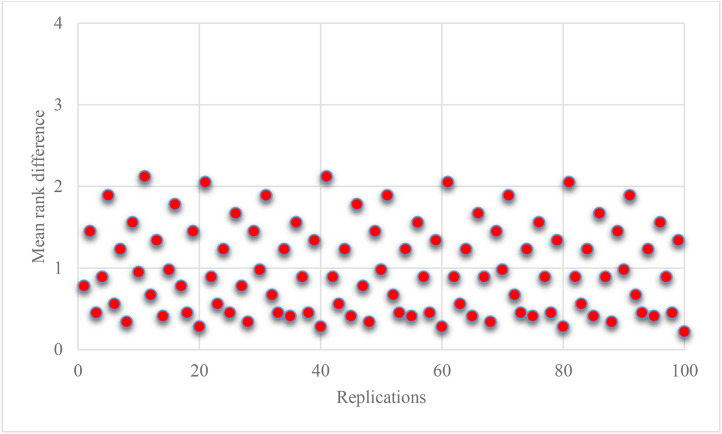
Scatter plot of mean rank difference of simulated alternatives under perturbed objective weights with higher priority to efficiency (100 Monte Carlo replications).

For each weight vector, the ranking model was solved, yielding 100 sets of alternative rankings along with the corresponding mean rank differences relative to the baseline solution (i.e., the ranking obtained with the original weights). To enhance interpretability, three scatter plots were repared, illustrating the distribution of the mean rank difference under each priority scenario together with the reference values of the original ranking (Fig 8, Fig 9, Fig 10).

Figures show that, given the presence of 35 alternatives and a ranking scale from 1 to 35, the observed mean rank differences (ranging from 0.98 to 3.65 units) do not appear substantial in the plots, indicating limited shifts in the final rankings. The clustered pattern of points around the baseline values reveals that the model exhibits acceptable stability in the ranking results across all three scenarios, with Scenario 3 (higher priority on efficiency compared to the other two objectives) demonstrating greater stability than the other scenarios. This structured validation confirms that changes in the importance weights under each scenario exert only a negligible influence on the alternative rankings, thereby attesting to the high reliability of the model.

Subsequently, a statistical analysis was performed to compare the distribution of simulated rank differences across the three scenarios (Table 5). For the economic-priority scenario, the mean rank difference was 2.74 with a standard deviation of 1.15, yielding a signal-to-noise ratio (mean/standard deviation) of 2.38. In the food-security-priority scenario, the mean rank difference was 3.65 with a standard deviation of 1.23 and a signal-to-noise ratio of 2.97. For the efficiency-priority scenario, the mean rank difference was 0.98 with a standard deviation of 0.56 and a signal-to-noise ratio of 1.75. According to quality-engineering criteria, signal-to-noise ratios above 1 were obtained for all scenarios, reflecting a small variation among the alternative rankings over the 100 replications and thus indicating high stability in the results. Consequently, it can be concluded that in every scenario the main ranking signal is markedly stronger than the noise induced by weight variations, confirming the high validity of the model.

**Table 5:** Statistical summary of model validation results.

Scenario	Mean Difference	Standard Deviation	Minimum	Maximum	Signal-to-Noise Ratio (SNR)
Economic Priority	2.74	1.15	0.72	5.12	2.38
Food Security Priority	3.65	1.23	1.72	5.56	2.97
Efficiency Priority	0.98	0.56	0.22	2.12	1.75

## 4. Discussion

The total yield of *A. bisporus* was significantly influenced by three factors: casing layer composition, nutritional supplement type, and the interaction between them. The treatment that produced the highest total yield combined the C1 casing with corn grit at 1000 g/m^2^ (S4) (see Section 3.1 and Fig 3c). This finding has two implications. First, it confirms the importance of casing quality. Second, it highlights a synergistic effect when a high-quality casing is combined with a balanced nutritional source. The superior performance of C1 casing is explained by its physical properties, including porosity, water-holding capacity, and aeration, which promote mycelial colonization and uniform pinhead formation [53,54,55]. In parallel, corn grit (S4) performed better than other supplements because it provides a readily available nutrient boost that does not disturb the physiological balance of the substrate or its microbiome.

Conversely, the lowest yield was associated with treatment C4S3 (Section 3.1). This pattern indicates that high-level protein supplementation, particularly with soybean meal, can exert strong negative effects on yield, effects which even casing soils of reasonable quality could not mitigate. The yield suppression from high-nitrogen supplements like soybean meal could be explained by several potential mechanisms: (1) possibly creating a nutritional imbalance in the substrate, promoting excessive vegetative mycelial growth on the casing surface at the expense of fructification [28,49]; (2) rapid microbial decomposition of nitrogenous compounds may release ammonia, which is toxic to mushroom mycelium [8]; and (3) increased competition from proliferating bacterial and mold populations, which might compromise oxygen penetration and weakens mycelial integrity [56]. These factors collectively explain the consistent yield decline observed across all casing types at the highest supplementation level (S3).

An equally important finding is the evident trade-off between fresh yield and dry matter content. While treatments with high soybean supplementation (e.g., S3) yielded the lowest fresh weights, they produced mushrooms with the highest dry matter percentage (exceeding 8%) and, consequently, the greatest absolute dry mass. This suggests that while high protein input may limit the number or size of fruiting bodies, it stimulates the synthesis of reserve materials and the development of denser tissues with lower moisture content [57,58]. This inverse relationship between yield and dry matter concentration, supported by earlier reports [59,60], has significant practical implications. High-yield treatments like C1S4 are ideal for the fresh market, whereas high dry-matter treatments like C3S3 may offer advantages for processing, extract production, or extended postharvest shelf-life.

Our results on the benefits of specific casing formulations and the risks of excessive supplementation align with and extend the existing literature. The critical role of casing physical structure is well-established [61,62], and the potential for non-peat materials to perform well has been noted [43]. Similarly, the yield-enhancing effect of balanced supplementation and the detrimental impact of nutrient overload are known [63,25]. However, this study strongly underscores the interactive nature of these factors. Maximum yield is only realized when a casing with optimal structure is paired with a compatible and balanced supplementation strategy. Supplements like corn grit, which may provide a more gradual nutrient release, appear synergistic with quality casings like C1. In contrast, highly soluble nitrogen sources like soybean meal can negate the benefits of good casing soils.

Beyond agronomic performance, this study provides critical insights for sustainable cultivation practices aligned with the principles of the circular economy and the urgent need for peat reduction. The significant yield obtained using the spent mushroom substrate (SMS)-based casing C5, particularly when paired with an effective supplement like corn grit, demonstrates a viable pathway for recycling this major by-product back into the production cycle [64]. This directly reduces waste and reliance on imported inputs, a key driver for circular practices, as demonstrated by Al-Dulayme et al. [65] on increased costs of imported casing soil and by Latif et al. [66] on using locally available materials like rice straw for oyster mushroom substrates. However, our results also suggest that SMS casings may not perform in isolation; their success is contingent upon balanced nutritional supplementation. This highlights that effective waste valorization requires integrated crop management, not merely substitution. Furthermore, the excellent performance of the peat-reduced or peat-free casing C1 is highly encouraging. It suggests that optimal physical properties—rather than peat content per se—are the key determinant of casing quality. This supports the ongoing shift within the industry towards structured, sustainable alternative materials and away from ecologically sensitive peatlands [67,43]. The use of corn grit, an agricultural milling by-product, as a superior supplement further reinforces the circular economy model by valorizing another secondary waste stream into a high-value input, mirroring the cost-saving logic seen in substrate optimization studies. Therefore, the most sustainable and productive system emerging from this study is not reliant on any single ‘magic bullet’ but on the synergistic combination of locally-sourced or recycled casing materials with strategically chosen, waste-derived nutritional supplements. This approach simultaneously addresses environmental goals (peat reduction, waste recycling) and economic imperatives (cost reduction, input security). Future research should focus on lifecycle assessments of these optimized formulations to quantify their full environmental and economic benefits, further solidifying the case for a circular model in mushroom cultivation.

Despite its benefits, recycled casing soil has important limitations. Recycled materials may exhibit greater variability in properties and, if sanitation is insufficient, repeated reuse could increase pathogen risks (e.g., Pseudomonas, Trichoderma) and accumulate non‑essential heavy metals (e.g., Cd, Pb). Although we applied leaching and fermentation, we did not monitor microbial succession or metal buildup across cycles, and our data cover only one cropping cycle [68]. More critically, the physiological mechanisms proposed in this study—including transporter saturation, ion antagonism, and ammonia toxicity—were inferred solely from yield and nutritional responses and lack direct molecular, enzymatic, or transcriptomic confirmation. Therefore, these interpretations should be viewed as hypothetical and require targeted experimental validation. These considerations do not diminish the value of recycling but underscore the need for routine quality monitoring, long‑term safety assessments across multiple cycles, and mechanistic research to ensure sustainable and safe commercial application.

The observed inverse relationship between yield and nutritional quality (Section 3.2.2, Fig 5) represents a fundamental physiological trade‑off. A dose‑response interpretation of these trade‑offs suggests the existence of distinct physiological thresholds that may govern mineral biofortification in *A. bisporus*. Elevating soybean meal from 1000 to 1500 g m ^−^ ^2^ elicited a proportional increase in FEI, consistent with a linear dose‑dependent regime. However, the concomitant decline in total yield and the elevated third‑flush contribution indicate that the nitrogen tolerance threshold was approached—and likely marginally exceeded—at the higher supplementation level. Mechanistically, this could be attributable to ammonia accumulation arising from rapid microbial deamination of excess proteinaceous substrates, which might impose osmotic and oxidative stress on the mycelium prior to fructification [19,25,48]. In marked contrast, corn grit exhibited a saturable pattern: increasing the application rate beyond 1000 g m ^−^ ² produced only a marginal increment in FEI, suggesting that the transport capacity for divalent cations (Mg^2+^  , Zn^2^ ^+^ , Cu^2^ ^+^ , Mn^2+^) approaches saturation already at the lower dose. This saturation may reflect finite transporter capacity or limited availability of intracellular metal-binding compounds (e.g., metallothioneins) in the mycelial compartment [69,60]. Importantly, the four minerals analyzed—Mg, Zn, Cu, and Mn—were selected precisely because of their well‑characterized roles as obligate enzyme cofactors (Mg^2+^ in ATPases and kinases, Zn^2+^ in superoxide dismutase and alkaline phosphatase, Cu^2+^ in cytochrome c oxidase and tyrosinase, Mn^2+^ in Mn‑SOD) and as structural elements (Zn^2+^ in zinc‑finger transcription factors regulating developmental gene expression) [70,60]. Consequently, the FEI serves not merely as a nutritional index but as a functional proxy for the metabolic saturation state of these cofactor‑dependent pathways. From an applied perspective, the choice of supplement dose must align with the grower’s primary objective: maximizing fresh yield favors corn grit at 1000 g m ^−^ ² (where transporter capacity remains unsaturated), whereas prioritizing mineral density justifies soybean meal at 1500 g m ^−^ ², despite the attendant yield penalty imposed by nitrogen excess. This compromise dictates that cultivation strategies must be aligned with specific production goals—maximizing yield or enhancing nutrient density.

Beyond dose‑response thresholds, the interactive dynamics among divalent and trace cations—competitive uptake, transporter saturation, and antagonistic cross‑talk—require mechanistic elucidation. In A. bisporus, Zn^2+^ and Cu^2^ ^+^ share low‑affinity, broad‑specificity transporters of the ZIP (ZRT/IRT‑related protein) family (e.g., AbZRT1, AbIRT1), whereas Mg^2+^ and Mn^2^ ^+^ are primarily acquired via MgtE‑type channels, CorA‑family transporters, and NRAMP‑family proteins [54]. Consequently, elevated substrate Zn^2^ ^+^ may potentially influence Cu^2^ ^+^ uptake through competition for shared transport pathways reported in fungi—a phenomenon that has been reported in other fungal species [71,72] and could also occur in *A. bisporus*. However, the simultaneous accumulation of Zn, Cu, and Mg under soybean meal supplementation (S3) argues against competitive exclusion in this context, instead suggesting that nitrogen‑rich organic matter enhances overall metal bioavailability via chelation or localized acidification. In contrast, the near‑saturation of mineral uptake for corn grit beyond 1000 g m ^−^ ² (Section 3.2.2) provides indirect evidence for finite transport capacity. Above this threshold, further increases in substrate Zn fail to proportionally elevate tissue Zn; instead, they may reduce Cu or Mn uptake through competition for metallochaperones (e.g., ATX1‑like proteins) or intracellular chelators such as metallothioneins [54]. Antagonistic interactions between Ca^2+^ and Mg^2^ ^+^ are particularly relevant here. All casing formulations contained 5% CaCO₃ (a common pH buffer and structural additive). Elevated Ca^2^ ^+^ is known to suppress Mg^2^ ^+^ uptake in filamentous fungi by competing for cation exchange sites on the mycelial cell wall and for MgtE‑type transporters [16]. The moderate Mg concentrations observed in C5 (20% Tima peat, 40% recycled soil, 5% CaCO_3_) may be partially associated with Ca–Mg interactions previously reported in fungi, although the present experiment was not designed to directly verify this mechanism. A similar competition between Ca^2+^ and Mn^2^ ^+^ has also been reported in basidiomycetes [53]. Collectively, these findings demonstrate that mineral biofortification in *A. bisporus* is governed not solely by substrate concentration but also by transporter saturation kinetics and inter‑ionic competition. Future studies should employ isotopic tracers (^65^Zn, ^64^Cu, ^25^Mg, ^54^Mn) coupled with transcriptomic profiling of ZIP, MgtE, NRAMP, and metallothionein genes to quantitatively resolve these interactive networks.

Corn, in various forms such as grits, husks, and silage, has been investigated as a supplement to improve the nutritional profile of compost [63]. Recent research by Eslamizadeh et al. [73] provides robust, quantitative evidence for this, demonstrating that corn grit supplementation significantly enhances both the yield and nutritional quality of *A. bisporus*. Their findings show that corn grit addition had a more pronounced effect than casing soil type on key parameters, notably increasing the total yield to a maximum of 36,669 g/m^2^ and elevating the protein content by up to 14.12% compared to the control. This direct improvement in nutritional profile is attributed to the rich composition of corn grit, which contains carbohydrates, proteins, and essential minerals such as Mg and Zn that facilitate superior mycelial metabolism and nutrient assimilation.The influence of the substrate and its supplement materials on the yield of the first two flushes, as demonstrated by Wang et al. [33], is thus clearly operationalized through corn grit’s role in boosting biological efficiency. Evaluation of edible mushroom yields in the first and second flushes under three types of substrates containing forest residues showed that the biological efficiency of the substrates varied from 61.89% to 81.01%; the work of Eslamizadeh et al. [73] corroborates that strategic supplement choice is a primary driver of such variation, with corn grit proving highly effective in optimizing substrate conversion into both biomass and valuable nutrients.

Finally, the safety and regulatory context of mineral biofortification in A. bisporus requires explicit consideration. All mineral concentrations achieved in this study—including Zn (up to 85 ppm dry weight), Cu (up to 45 ppm), and Mg (up to 1200 ppm)—remain well within the upper tolerable intake levels established by the European Food Safety Authority (EFSA) and the World Health Organization/Food and Agriculture Organization (WHO/FAO) [74,75,76,77]. For a typical 100 g serving of fresh mushrooms (assuming 90% moisture), the calculated Zn intake (~8.5 mg) falls within the recommended dietary allowance (8‑11 mg for adults) and below the EFSA tolerable upper intake level of 25 mg day ⁻ ¹ [74,78]. Similarly, Cu intake (~4.5 mg) approaches but does not exceed the EFSA upper limit of 5 mg day ⁻ ¹ [76], while Mg intake (~120 mg) remains below the supplemental upper limit of 250 mg day ⁻ ¹ [75]. The Food Safety and Standards Authority of India (FSSAI) and Codex Alimentarius do not set specific maximum limits for Zn, Cu, or Mg in edible mushrooms due to their low toxicity profile compared to heavy metals such as Cd, Pb, or As [68]. From an ethical standpoint, agronomic biofortification via casing soil and compost supplementation is preferable to post‑harvest fortification because it preserves the natural food matrix. Nevertheless, repeated use of recycled casing soil should be accompanied by periodic monitoring of non‑essential trace elements (e.g., Cd, Pb) according to national standards [79].

## 5. Conclusion

This study addresses the fundamental challenge of balancing economic profitability, nutritional quality, and production efficiency in sustainable button mushroom cultivation, providing a decision-making framework through multi-objective goal programming optimization. Our results demonstrate that a synergistic use of recycled casing soils with corn grit supplementation establishes a robust, agronomically sound model for commercial cultivation. Specifically, the combination of a casing soil containing 40% recycled material with 1500 g/m^2^ corn grit is identified as a highly effective strategy. This formula delivered superior economic return while maintaining strong yield efficiency in the first two flushes and preserving nutritional value. The higher application rate of corn grit further enhanced operational efficiency by concentrating yield in the initial flushes, which can translate into significant energy savings and a shorter production cycle. While imported peat-based substrates with soybean meal supplementation can maximize certain nutritional content metrics, our analysis—supported by a comprehensive economic feasibility assessment—confirms that their associated high costs often compromise the economic sustainability for commercial growers. Critically, the goal programming model developed here was subjected to a rigorous Monte Carlo simulation and sensitivity analysis. This validation confirms its stability and robustness against input variability, demonstrating that its recommendations are not artifacts of experimental noise. Therefore, the model provides a robust and data-driven decision-support tool for growers. It enables them to systematically evaluate trade-offs and identify optimal cultivation formulas that align with their specific, weighted priorities for economic, nutritional, and efficiency goals. By integrating scientific data with practical optimization, this work provides a clear pathway toward a more sustainable, profitable, and resilient mushroom industry.

## Supporting information

S1 Text GAMS code for the goal programming (GP) model implementation. The code includes the complete mathematical formulation with sets, parameters, binary variables, constraints (assignment, unique rank, target reach), and the objective function for minimizing total deviation. The model is solved using a mixed-integer programming (MIP) solver to rank 35 cultivation alternatives under multiple priority weight scenarios.
(PDF)

S1 Table Analysis of variance (ANOVA) for nutritional components of white button mushroom under different casing soils and nutritional supplements. This table includes complete ANOVA results (degrees of freedom, sum of squares, mean squares, F-values, and p-values) for crude protein, crude fiber, magnesium (Mg), copper (Cu), zinc (Zn), and manganese (Mn) across seven casing formulations and five supplement levels.
(PDF)

S2 Table Nutritional composition (mean ± SE) of white button mushrooms across 35 cultivation alternatives. Data are presented for magnesium (Mg), copper (Cu), zinc (Zn), manganese (Mn), crude fiber, and crude protein on a dry weight basis. Each value represents the mean of three independent replicates (n = 3 per alternative) with standard error.
(PDF)
