# Supplementary material for: Economic, nutritional, and efficiency assessment of recycled casing soil and compost nutritional supplements for sustainable white button mushroom cultivation
Source: PLoS One. 2026 Aug 13;21(8):e0337146. doi: 10.1371/journal.pone.0337146 (PMC13472422; doi:10.1371/journal.pone.0337146)
Supplement: Supplementary file 1 [file pone.0337146.s001.pdf]

S1 Text. GAMS code for the goal programming (GP) model implementation. The code includes the complete mathematical formulation with sets, parameters, binary variables, constraints (assignment, unique rank, target reach), and the objective function for minimizing total deviation. The model is solved using a mixed-integer programming (MIP) solver to rank 35 cultivation alternatives under multiple priority weight scenarios.

https://doi.org/10.1371/journal.pone.0337146.s001

```
***********************************************************
Sets
        a alternatives /a1*a35/
        r ranks/1*35/;
***********************************************************
**Data
***********************************************************
Parameter    pr(a)   Profit of alternatives (USD per m2);
Parameter    fe(a)   Nutritional index (per m2);
Parameter    ry(a)   ratio of third yield to total yield (% per m2);
Parameter Rank_pr(a), Rank_fe(a), Rank_ry(a);
Loop (a,
   Rank_pr(a) = 1 + sum(i$(pr(i) > pr(a)), 1);
   Rank_fe(a) = 1 + sum(i$(fe(i) > fe(a)), 1);
   Rank_ry(a) = 1 + sum(i$(ry(i) < ry(a)), 1);
);
display Rank_pr, Rank_fe, Rank_ry;
Parameter Target_Rank(a);
Target_Rank(a) = (50*Rank_pr(a) + 50*Rank_fe(a) + 200*Rank_ry(a))/300;
display Target_Rank;
*************************************************************
**Variables
*************************************************************
Positive variables
   dev_plus(a)
   dev_minus(a) ;
Binary variables   h(a,r);
```

```
Variables     total_deviation;
****************************************************************
**Equations
****************************************************************
Equations
assign_rank(a)
unique_rank(r)
rank_definition(a)
objective;
assign_rank(a)..        sum (r, h(a,r)) =e= 1;
unique_rank(r)..        sum (a, h(a,r)) =e= 1;
rank_definition(a)..   sum (r, h(a,r) * ord(r)) =e= Target_Rank(a) + dev_plus(a) - dev_minus(a);
objective..                  total_deviation =e= sum (a, dev_plus(a) +dev_minus(a));
****************************************************************
**Solving method
****************************************************************
Model rank_optimization /all/;
Solve rank_optimization using mip minimizing total_deviation;
parameter rankt(a);
rankt(a)=sum (r, h.l(a,r) * ord(r));
display rankt;
****************************************************************
```
