# Supplementary material for: Economic, nutritional, and efficiency assessment of recycled casing soil and compost nutritional supplements for sustainable white button mushroom cultivation
Source: PLoS One. 2026 Aug 13;21(8):e0337146. doi: 10.1371/journal.pone.0337146 (PMC13472422; doi:10.1371/journal.pone.0337146)
Supplement: Supplementary file 2 [file pone.0337146.s002.pdf]

S1 Table. Analysis of variance (ANOVA) for nutritional components of white button mushroom under different casing soils and nutritional supplements. This table includes complete ANOVA results (degrees of freedom, sum of squares, mean squares, F-values, and p-values) for crude protein, crude fiber, magnesium (Mg), copper (Cu), zinc (Zn), and manganese (Mn) across seven casing formulations and five supplement levels.

https://doi.org/10.1371/journal.pone.0337146.s002

Crude Protein

Source	DF	Adj SS	Adj MS	F-Value	P-Value
Replication	2	0.7493	0.37466	1217.46	0.000
casing soil	6	0.2510	0.04183	135.93	0.000
Supplement	4	22.0774	5.51934	17935.33	0.000
casing soil*Replication	12	0.0004	0.00003	0.11	1.000
casing soil*Supplement	24	0.6625	0.02760	89.70	0.000
Error	56	0.0172	0.00031		
Total	104	23.7578			

Crude Fiber

Source	DF	Adj SS	Adj MS	F-Value	P-Value
Replication	2	110.512	55.2560	55.61	0.000
casing soil	6	2.555	0.4258	0.43	0.857
casing soil*Replication	12	9.082	0.7569	0.76	0.686
Supplement	4	17.150	4.2875	4.32	0.004
casing soil*Supplement	24	50.852	2.1188	2.13	0.010
Error	56	55.643	0.9936		
Total	104	245.794			

Cu

Source	DF	Adj SS	Adj MS	F-Value	P-Value
Replication	2	97.25	48.625	2572.71	0.000
Casing soil	6	1654.88	275.814	14593.10	0.000
Casing soil*Replication	12	14.16	1.180	62.42	0.000
Supplement	4	121.16	30.290	1602.64	0.000
Casing soil*Supplement	24	2.53	0.105	5.58	0.000
Error	56	1.06	0.019		
Total	104	1891.04			

Mg

Source	DF	Adj SS	Adj MS	F-Value	P-Value
Replication	2	277637	138818	80076.82	0.000
Casing soil	6	37648	6275	3619.56	0.000
Casing soil*Replication	12	326	27	15.66	0.000
Supplement	4	10837	2709	1562.81	0.000
Casing soil*Supplement	24	414	17	9.95	0.000
Error	56	97	2		
Total	104	326959			

Mn

Source	DF	Adj SS	Adj MS	F-Value	P-Value
Replication	2	14.272	7.1360	2675.35	0.000
Casing soil	6	405.748	67.6247	25353.04	0.000
Casing soil*Replication	12	3.468	0.2890	108.36	0.000
Supplement	4	13.678	3.4194	1281.97	0.000
Casing soil*Supplement	24	3.725	0.1552	58.19	0.000
Error	56	0.149	0.0027		
Total	104	441.040			
