# Supplementary material for: Economic, nutritional, and efficiency assessment of recycled casing soil and compost nutritional supplements for sustainable white button mushroom cultivation
Source: PLoS One. 2026 Aug 13;21(8):e0337146. doi: 10.1371/journal.pone.0337146 (PMC13472422; doi:10.1371/journal.pone.0337146)
Supplement: Supplementary file 3 [file pone.0337146.s003.pdf]

S2 Table. Nutritional composition (mean ± SE) of white button mushrooms across 35 cultivation alternatives. Data are presented for magnesium (Mg), copper (Cu), zinc (Zn), manganese (Mn), crude fiber, and crude protein on a dry weight basis. Each value represents the mean of three independent replicates (n = 3 per alternative) with standard error.

https://doi.org/10.1371/journal.pone.0337146.s003

Alternatives	Mg	Cu	Zn	Mn	Crude Fiber	Crude Protein
a1	534.50 ± 15.761	16.05 ± 0.473	46.13 ± 1.360	7.08 ± 0.209	10.69 ± 1.180	0.69 ± 0.025
a2	544.70 ± 16.064	17.66 ± 0.426	51.25 ± 1.209	7.92 ± 0.184	10.35 ± 0.357	0.80 ± 0.023
a3	554.90 ± 16.368	20.06 ± 0.280	59.45 ± 0.384	8.75 ± 0.083	11.16 ± 0.378	0.91 ± 0.022
a4	549.80 ± 16.672	16.85 ± 0.245	53.30 ± 0.351	7.50 ± 0.077	12.27 ± 0.378	0.67 ± 0.024
a5	565.10 ± 16.975	18.46 ± 0.210	56.38 ± 0.329	7.27 ± 0.070	11.84 ± 0.348	0.76 ± 0.026
a6	544.70 ± 17.279	14.45 ± 0.175	41.00 ± 0.307	6.25 ± 0.064	9.63 ± 0.388	0.62 ± 0.024
a7	554.90 ± 17.583	16.05 ± 0.140	46.13 ± 0.275	7.08 ± 0.058	10.59 ± 0.373	0.78 ± 0.023
a8	565.10 ± 16.064	18.46 ± 0.521	54.33 ± 1.511	7.92 ± 0.233	9.84 ± 0.383	0.91 ± 0.028
a9	560.00 ± 16.368	15.25 ± 0.473	49.20 ± 1.360	6.67 ± 0.209	10.48 ± 0.392	0.64 ± 0.029
a10	575.30 ± 16.672	16.85 ± 0.315	55.35 ± 0.439	7.33 ± 0.096	11.98 ± 0.395	0.79 ± 0.030
a11	554.90 ± 16.975	9.50 ± 0.280	13.04 ± 0.406	2.83 ± 0.090	10.22 ± 0.381	0.67 ± 0.030
a12	565.10 ± 17.279	10.68 ± 0.245	14.90 ± 0.384	3.26 ± 0.098	10.67 ± 0.355	0.79 ± 0.028
a13	575.30 ± 17.583	12.46 ± 0.210	17.51 ± 0.362	3.70 ± 0.077	10.32 ± 0.389	0.93 ± 0.030
a14	570.20 ± 17.886	10.09 ± 0.175	16.02 ± 0.329	3.64 ± 0.070	10.78 ± 0.384	0.67 ± 0.029
a15	585.50 ± 18.023	11.28 ± 0.592	17.13 ± 0.439	3.39 ± 0.233	10.63 ± 0.413	0.76 ± 0.033
a16	565.10 ± 18.177	8.31 ± 0.544	11.92 ± 1.602	2.61 ± 0.109	10.22 ± 0.364	0.62 ± 0.033
a17	575.30 ± 16.792	9.50 ± 0.367	13.78 ± 0.516	3.04 ± 0.103	10.30 ± 0.382	0.78 ± 0.034
a18	585.50 ± 17.279	11.28 ± 0.332	16.39 ± 0.483	3.48 ± 0.111	10.06 ± 0.372	0.90 ± 0.033
a19	580.40 ± 17.583	8.90 ± 0.298	14.90 ± 0.439	2.83 ± 0.09	10.76 ± 0.391	0.68 ± 0.032
a20	595.70 ± 18.023	10.09 ± 0.262	15.64 ± 0.417	3.17 ± 0.083	10.34 ± 0.379	0.74 ± 0.033
a21	575.30 ± 18.177	7.12 ± 0.227	11.17 ± 0.384	2.39 ± 0.077	9.41 ± 0.393	0.63 ± 0.033
a22	585.50 ± 16.512	8.31 ± 0.497	13.04 ± 1.571	3.33 ± 0.221	9.59 ± 0.454	0.78 ± 0.025
a23	595.70 ± 16.517	10.09 ± 0.450	14.90 ± 1.451	3.76 ± 0.196	10.58 ± 0.388	0.87 ± 0.026
a24	590.60 ± 16.821	7.72 ± 0.298	13.78 ± 0.472	2.61 ± 0.107	10.76 ± 0.399	0.68 ± 0.026
a25	605.90 ± 17.125	8.90 ± 0.262	14.53 ± 0.439	2.96 ± 0.083	10.31 ± 0.398	0.73 ± 0.026
a26	585.50 ± 17.428	5.93 ± 0.227	10.43 ± 0.406	2.17 ± 0.077	10.50 ± 0.398	0.63 ± 0.027
a27	595.70 ± 17.732	7.12 ± 0.193	12.29 ± 0.384	2.61 ± 0.070	10.52 ± 0.396	0.78 ± 0.025
a28	611.00 ± 18.023	8.90 ± 0.157	14.15 ± 0.351	3.04 ± 0.073	10.26 ± 0.399	0.90 ± 0.027
a29	600.80 ± 18.177	6.53 ± 0.544	13.04 ± 1.664	2.39 ± 0.214	10.71 ± 0.438	0.65 ± 0.029
a30	616.10 ± 18.468	7.72 ± 0.497	13.78 ± 1.632	2.74 ± 0.216	9.15 ± 0.443	0.76 ± 0.030
a31	595.70 ± 17.279	4.75 ± 0.332	9.31 ± 0.505	1.96 ± 0.100	10.09 ± 0.393	0.63 ± 0.030
a32	605.90 ± 17.583	5.93 ± 0.298	11.17 ± 0.461	2.39 ± 0.094	10.38 ± 0.382	0.80 ± 0.028
a33	616.10 ± 17.886	7.72 ± 0.262	13.04 ± 0.428	2.83 ± 0.087	10.62 ± 0.381	0.91 ± 0.029
a34	611.00 ± 18.177	5.34 ± 0.227	11.92 ± 0.406	2.47 ± 0.081	10.79 ± 0.338	0.71 ± 0.029
a35	626.30 ± 18.468	6.53 ± 0.193	12.66 ± 0.373	2.52 ± 0.074	9.02 ± 0.334	0.73 ± 0.029
