# Supplementary material for: Economic, nutritional, and efficiency assessment of recycled casing soil and compost nutritional supplements for sustainable white button mushroom cultivation
Source: PLoS One. 2026 Aug 13;21(8):e0337146. doi: 10.1371/journal.pone.0337146 (PMC13472422; doi:10.1371/journal.pone.0337146)
Supplement: Supplementary file 7 [file pone.0337146.s007.docx]

**Response to Reviewers**

Manuscript ID: PONE-D-25-59546R2

Title: Economic, Nutritional, and Efficiency Assessment of Recycled Casing Soil and Compost Nutritional Supplements for Sustainable White Button Mushroom Cultivation

Journal: PLOS ONE

**Dear Editor and Reviewer 2,**

We sincerely thank the reviewer for the constructive comments on our revised manuscript. We are pleased that the major concerns have been addressed satisfactorily. Below we respond to each remaining minor revision point by point.

**Response to Reviewer 2**

Comment 1:

"In the discussion section (Pages 26–30), several explanations regarding transporter activity, enzymatic regulation and nutrient uptake mechanisms are presented too definitively despite the absence of molecular, enzymatic or metabolomic validation. These statements should be revised as probable physiological explanations rather than experimentally confirmed mechanisms."

**Response:** We agree and have carefully revised the Discussion section accordingly. All mechanistic interpretations—including transporter activity, ion antagonism, and ammonia‑induced toxicity—are now presented as plausible physiological explanations or testable hypotheses rather than confirmed mechanisms. Definitive language such as "reflects" has been replaced with more cautious phrasing such as "may be partially explained by", "is consistent with", and "suggests". We have also explicitly stated that these interpretations require direct molecular, enzymatic, or transcriptomic validation in future studies.

Comment 2:

"Although regulatory discussion has been added by the authors, but I still recommend to add possible microbial contamination risks, heavy metal accumulation concerns associated with recycled casing soil. A brief limitation statement should be included in the discussion section."

**Response:** We have added a dedicated limitation paragraph in the Discussion section that explicitly addresses: (i) potential microbial contamination risks (e.g., Pseudomonas spp., Trichoderma spp.) with repeated reuse of recycled casing soil; (ii) possible gradual accumulation of non‑essential heavy metals (e.g., Cd, Pb) over multiple cycles; (iii) the single‑cycle scope of our data; and (iv) the hypothetical nature of our proposed physiological mechanisms. This paragraph has been inserted following the circular economy benefits discussion.

Comment 3:

"The authors can avoid using terms such as 'innovative framework,' 'methodological breakthrough,' and similar promotional terms."

**Response:** We have removed all promotional and overly assertive language throughout the manuscript. For example, "Our primary innovation is" has been changed to "This study presents"; "methodological breakthrough" has been replaced with "approach"; and "novel decision‑making framework" has been revised to "decision‑making framework". Similar neutralizations have been applied consistently across the Abstract, Introduction, and Conclusion.

Comment 4:

"Some sections, particularly the Goal Programming methodology and Discussion, remain repetitive. Avoid repetitions."

**Response:** We have eliminated redundancies as follows. First, the detailed GP explanation in the Introduction has been condensed to a brief 2‑line summary that refers readers to Section 2.2 for the full formulation. Second, numerical yield values (e.g., 36,669 g/m²) have been removed from the Discussion and replaced with references to Section 3.1 and Fig. 3c. Third, the repeated comparison between C1S4 and C1S3 in the Discussion has been streamlined with a concise reference to Section 3.2.2 and Fig. 5.

Comment 5:

"Check all in‑text citations with full references."

**Response:** We have meticulously cross‑checked every in‑text citation against the reference list. All newly added references are correctly numbered, and no retracted papers were identified. All citations now appear in the correct order and format according to PLOS ONE guidelines.

**Summary of Revisions**

All changes described above are clearly highlighted in the "Revised Manuscript with Track Changes" file. An unmarked clean version of the manuscript is also provided. In summary: mechanistic interpretations in the Discussion have been softened to hypothetical explanations; a new limitation paragraph addresses microbial and heavy metal risks; promotional language has been removed; redundancies between sections have been eliminated; and all in‑text citations have been verified against the reference list.

We believe the revised manuscript now fully addresses all remaining concerns and hope it is acceptable for publication in PLOS ONE.

Yours sincerely,

Corresponding Author
