# Supplementary material for: Economic, nutritional, and efficiency assessment of recycled casing soil and compost nutritional supplements for sustainable white button mushroom cultivation
Source: PLoS One. 2026 Aug 13;21(8):e0337146. doi: 10.1371/journal.pone.0337146 (PMC13472422; doi:10.1371/journal.pone.0337146)
Supplement: Supplementary file 8 [file pone.0337146.s004.docx]

- The introduction is unusually long, overly descriptive, and contains repeated justifications.
- Why not use multi-criteria methods like:
- TOPSIS, AHP, MOORA, Multi-objective linear programming directly (without ranking)?
- The figures need Improvement as many graphs lack error bars and Axis labels are not visible or too small.
- The result section reads like a raw extraction from analyses. More synthesis is needed.
- Several sentences are very long, contain redundancies, or use overly formal/unnecessary phrasing.
- Rewrite your Abstract
- Improve the discussion
