# Supplementary material for: Economic, nutritional, and efficiency assessment of recycled casing soil and compost nutritional supplements for sustainable white button mushroom cultivation
Source: PLoS One. 2026 Aug 13;21(8):e0337146. doi: 10.1371/journal.pone.0337146 (PMC13472422; doi:10.1371/journal.pone.0337146)
Supplement: Supplementary file 9 [file pone.0337146.s005.docx]

**Dear Editors and Reviewers,**

We hope this message finds you well. We are pleased to submit the revised version of our manuscript entitled **“Economic, Nutritional, and Efficiency Assessment of Recycled Casing Soil and Compost Nutritional Supplements for Sustainable White Button Mushroom Cultivation.”**

We have carefully considered all the reviewers' comments and have revised the manuscript accordingly. For clarity, each reviewer's comments have been addressed in the following color-coded sections:

- **Reviewer 1:** Yellow highlights
- **Reviewer 2:** Green highlights
- **Reviewer 3:** Yellow & Green highlights

We believe that the revisions have significantly strengthened the manuscript, and we are grateful for the insightful and constructive feedback provided by the reviewers.

Thank you for your time and consideration.

**Reviewer #1:**

The manuscript presents a well-designed and methodologically sound study addressing sustainable cultivation strategies for Agaricus bisporus using a goal programming (GP) framework. The integration of field experiments with optimization modeling is commendable and provides both scientific and practical insights into sustainable mushroom production systems. The topic is timely and aligns with global sustainability and circular economy goals. However, several minor issues related to structure, clarity, and presentation should be addressed to enhance readability and overall impact.
1. The abstract and conclusion should be revised to provide a technical glimpse of overall study.
2. Throughout the manuscript, there is extensive use of apostrophe s ('s) which pertains to a story telling method rather than a technically proficient language.

**Response:**

We sincerely thank Reviewer #1 for their positive assessment of the study's design, methodology, and relevance to sustainability goals. We are pleased that the integration of field experiments with optimization modeling was found to be commendable.

We have carefully addressed the two specific points raised to enhance the manuscript's technical proficiency and clarity:

1. Revision of abstract and conclusion: As suggested, we have thoroughly revised both the Abstract and the Conclusion sections. The revisions ensure these sections provide a clear, concise, and technically precise overview of the study's objectives, methodology, key findings, and implications, moving from a descriptive to a more analytical summary.

2. Correction of language style: We acknowledge the reviewer's point regarding the informal use of the apostrophe *'s* (possessive/genitive case) in a narrative style. We have performed a comprehensive edit throughout the entire manuscript to replace this colloquial construction with more formal and precise technical language. For instance, phrases like *"the study's goal"* have been revised to *"the goal of this study"* or *"the study objective."* This revision ensures the manuscript adheres to the standard, objective prose expected in scientific literature.

We believe these revisions have strengthened the manuscript's structure, clarity, and professional tone. We thank the reviewer again for their constructive feedback.

**Reviewer #2:**

I had critically gone through the MS and observed the following:

The concept of MS is good, and the combination of experimental and decision-optimization approaches is the need of the hour in mushroom cultivation. However, the manuscript requires substantial revisions before it can be considered for publication.

**Comment 1:** The introduction contains an excessive amount of general information about mushrooms, sustainability, and the sugarcane sector in Iran. The objectives of the study are missing, and the authors are advised to include a paragraph explaining how the present study is unique and novel compared to existing ones. Focus more sharply on the knowledge gap.

**Response:**
Thank you for your insightful feedback regarding the Introduction section. We have carefully revised it to address your concerns by focusing more sharply on the knowledge gap, clarifying the novelty of our study, and explicitly stating the research objectives. The key changes are as follows:

The paragraph on the sugarcane industry in Iran has been removed, and general background information on mushrooms has been condensed to maintain a sharper focus on the research problem.

A new paragraph has been added towards the end of the Introduction to clearly state the research gap: the lack of an integrated framework for simultaneously optimizing the interactions between recycled casing soils and nutritional supplements under multiple objectives (economic, nutritional, and efficiency), and for merging empirical agronomic data with multi-criteria decision modeling. This paragraph explicitly presents our two-phase experimental-modeling approach as the novel contribution addressing this gap.

Immediately following the novelty statement, a dedicated paragraph now lists the three specific research objectives in a numbered format:

Objective 1: To evaluate the effects of casing soils and supplements via field experiments.

Objective 2: To develop a Goal Programming model for simultaneous optimization of the three competing goals.

Objective 3: To identify the optimal strategy through sensitivity analysis and provide a practical decision-support tool.

These revisions ensure that the scientific justification, novelty compared to prior work, and clear research aims are now prominently and concisely highlighted in the Introduction.

**Comment 1.1:** How casing soil and supplement interactions are not yet optimized under multi-objective settings.

**Response:**

Thank you for your insightful comment regarding the optimization of casing soil and supplement interactions under multi-objective settings.

In this study, we explicitly modeled and optimized the interaction between casing soil formulations and nutritional supplements within a multi-objective Goal Programming (GP) framework. Each of the 35 treatment combinations (7 casing soils × 5 supplements) was evaluated as an independent alternative and ranked based on three objectives: economic profit, nutritional index, and third-flush yield ratio.

Through the GP model, we not only ranked all alternatives but also conducted sensitivity analysis (Table 3 and Fig. 7) to demonstrate how different priority weightings (economic, nutritional, efficiency) lead to distinct optimal combinations of casing soil and supplement. For example:

When economic profit is prioritized, the optimal combination is C5 (20% Tima peat + 40% recycled soil + bagasse) with S4 (corn grit at 1000 g/m²).

When nutritional quality is emphasized, C2 (95% Tima peat) with S5 (corn grit at 1500 g/m²) performs best.

When efficiency is prioritized, C5 with S5 (corn grit at 1500 g/m²) is selected.

Thus, the interaction between casing soil and supplement is systematically optimized under multi-objective preferences, and the model provides a decision-support tool for selecting the best combination based on grower priorities.

**Comment 1.2:** Why is integrating agronomic data with GP modeling scientifically justified?

**Response:**

The integration of agronomic experimental data with Goal Programming (GP) modeling is scientifically justified for the following reasons:

1. Bridging the gap between agronomic research and practical decision-making: While field experiments provide valuable data on crop performance under different treatments, they do not inherently resolve trade-offs between conflicting objectives (e.g., profit vs. nutrition vs. sustainability). GP provides a structured framework to incorporate these experimental results into a multi-objective decision model, enabling the identification of optimal strategies that balance competing priorities.
2. Handling multi-dimensional agricultural challenges: Sustainable agriculture inherently involves multiple and often conflicting goals. GP allows for the simultaneous optimization of economic, nutritional, and efficiency objectives, which cannot be achieved through conventional univariate statistical analysis alone.
3. Enhancing real-world applicability: By embedding real agronomic data into the GP model, the results are grounded in empirical evidence rather than theoretical assumptions. This increases the practical relevance and adoptability of the recommendations for farmers and agribusinesses.
4. Supporting evidence-based policy and farm management: The combined approach provides a transparent and reproducible method for evaluating sustainable practices under different scenarios (e.g., changing input prices, environmental regulations, or consumer preferences).
5. Methodological synergy: The experimental design ensures robust and statistically valid data, while GP translates these data into actionable insights through mathematical optimization. This synergy strengthens both the agronomic and decision-science contributions of the study.

In summary, the integration is not merely a technical exercise but a scientifically coherent approach to addressing complex, real-world problems in sustainable agriculture through a combination of empirical rigor and operational research.

**Comment 2:** Move regional sugarcane industry details to the Discussion or as a brief contextual note.

**Response:**

We thank the reviewer for this constructive suggestion regarding the flow of the manuscript. In our final revision, we have removed the detailed description of the regional sugarcane industry from the Introduction section entirely.

Upon careful reconsideration, we determined that while this context is interesting, its level of detail was not essential for framing the core research problem of optimizing mushroom cultivation substrates. Retaining it, even in a condensed form within the Discussion, could distract from the primary focus on our experimental results and their direct agronomic and economic implications.

Therefore, to maintain a sharp, concise, and focused narrative throughout the paper, we have opted to omit this specific regional industrial detail.

**Comment 3:** The manuscript mentions ANOVA and Tukey’s test, but key elements are missing, including Mean ± SE values in figures or tables, whether data were transformed (especially percentage and ratio data), and the Number of samples for nutritional analysis.

**Response:**

Thank you for this important methodological comment. We have revised the manuscript to include the requested details.

Mean ± SE values: The values in Figures 3 a, b and Tables A2 have been updated to include Mean ± Standard Error (SE). The error bars in these figures now explicitly represent the SE. Corresponding figure captions have been revised to state, Error bars represent the standard error of the mean (SE).

Data transformation: The percentage data (e.g., yield share per flush, proportion of second-grade mushrooms) and ratio data (third-flush yield ratio) were not transformed prior to ANOVA, as their residuals met the assumptions of normality and homogeneity of variance. This clarification has been added to Section 2.1: "The assumptions of normality and homogeneity of variances for ANOVA were checked and met for all variables, including percentage and ratio data; therefore, no data transformation was applied."

Sample size and preparation for nutritional analysis: The number of samples and the detailed preparation protocol for the nutritional and mineral content analysis have been clearly stated in the revised Section 2.1.10. These additions ensure full transparency and reproducibility of our statistical and analytical procedures. The relevant sections (2.1, 2.1.10, and figure captions) have been updated accordingly in the manuscript.

*** It is important to note that there is a distinction between statistical models and mathematical optimization models such as goal programming (GP). The GP model is a deterministic optimization framework, not a statistical estimation model. Its purpose is to find the optimal ranking of alternatives given a specific set of inputs (experimental data, costs, prices) and constraints. Therefore, concepts such as standard error (SE) or confidence intervals, which quantify uncertainty in estimated parameters of a statistical relationship, are not applicable to the final GP solution (the ranking itself). Uncertainty and robustness in optimization models are instead assessed through sensitivity analysis on key model inputs. In our study, this was comprehensively addressed by varying the objective weights (Table 3 and Fig. 7). This analysis shows how the optimal ranking changes with different decision-maker priorities, effectively demonstrating the model's stability and the robustness of the top-ranked alternatives to changes in these parameters. The core solution from GAMS is the single, mathematically optimal ranking for a given set of weights and data.

**Comment 4:** Authors are suggested to provide a full ANOVA table with all p-values and also clearly define the Main plot error and the Subplot error.

**Response:**

 We have now provided a complete ANOVA table for the total yield data in the main text, which includes all degrees of freedom (df), sum of squares, F-values (implicitly via significance stars), and exact p-values as requested. Furthermore, we have clearly defined the error terms for the split-plot design directly in the table caption and/or the accompanying text.

Specifically:

Main Plot Error (Error a): This is the error term used to test the effect of the casing soil factor.

Subplot Error (Error b): This is the error term used to test the effect of the supplement factor and the casing soil × supplement interaction.

Additionally, in the Supplementary Materials (Appendix A2), we have provided the full set of detailed ANOVA tables for all analyzed nutritional components (protein, fiber, Cu, Mg, Mn), following the same structure with all relevant statistical parameters.

We believe these additions provide full transparency regarding the statistical analysis and meet the reviewer's request.

**Comment 5:** Add sample size (n) for all parameters, including mineral analysis.

**Response:**

Thank you for highlighting this important point. We have now explicitly stated the sample size (n) for all measured parameters throughout the Materials and Methods section (Section 2).

Specifically, for the mineral element analysis, we have added the following clarification in Section 2.1.10:

"For mineral element analysis, the sample size was n = 3. Each replicate consisted of 4 similarly-sized mushrooms, which were dried, powdered, and homogenized together to form a single composite sample per replicate."

For clarity, we also confirm that the same sample size (n = 3) was applied consistently across all other measured parameters (yield, dry matter, protein, fiber, etc.), with each replicate representing an independent experimental unit (a designated tray/m² within the randomized block design). This information is now clearly stated in the relevant methodological subsections.

**Comment 6 & 7:**

6. Add measures of variation (SE/SD) to Fig. 3, 5, and 6.
7. Figures lack clarity and essential information. Figures 3–6 do not include: Error bars, Sample sizes, Statistical groupings (only letters without explanation), Units on axes for nutritional index and ratios. Rectify these mistakes.

**Response:**

We have revised Figures 3, 5, and 6 to incorporate all requested elements:

1. Addition of Error Bars: All bar graphs and data points in the mentioned figures now include error bars representing the Standard Error of the Mean (SE). The exact SE values are also provided in the corresponding supplementary data tables.
2. Clarification of Sample Size (n): The sample size (n = 3 independent replicates per treatment) is now explicitly stated in the revised captions for Figures 3, 5, and 6. This information was also added to the Materials and Methods section (2.1.10) as per our previous response.
3. Explanation of Statistical Groupings: The figure captions now contain a clear legend explaining the meaning of the lowercase letters above the bars/data points. For example: *"*Different lowercase letters above bars indicate statistically significant differences between treatments according to Tukey's HSD test (p < 0.05)."
4. Axis Labels and Units:
   - All axes now have complete and clear labels.
   - For axes representing nutritional indices (which are unitless, calculated from standardized and weighted scores), the axis label has been updated to: "Nutritional Index (Unitless)" to prevent any ambiguity.
   - For axes representing ratios (e.g., third-flush yield ratio), the label now reads: "Yield Ratio (Third Flush / Total)" or equivalent, making the calculation and its unitless nature explicit.
   - All other axes (e.g., Yield in g/m², Mineral Content in mg/kg DW) display their appropriate, unambiguous units.

The revised figures now provide a complete visual summary of the data, including central tendency (mean), precision (SE error bars), sample size (stated in caption), statistical differences (grouping letters with explanation), and clear, unambiguous axis labels with units. We believe these comprehensive changes fully address the reviewer's concerns and significantly enhance the interpretability and scientific rigor of the graphical data presentation.

**Comment 8 & 9:**

8. The manuscript does not explain:

• Why 1000 and 1500 g/m² were chosen for soybean/corn.

• Why do recycled soil fractions follow exactly the chosen percentages?

• Whether C5 and C6 formulations were based on previous optimization trials.

9. The authors are advised to cite prior work or provide preliminary results supporting chosen levels.

**Response:**

Thank you for raising these important points regarding the experimental design. We have revised the manuscript to include clear justifications for the chosen supplement rates and casing soil formulations, as detailed below.

1. Regarding the selection of 1000 and 1500 g/m² for soybean and corn meals:

These amounts were used as supplements during the compost preparation stage. The common commercial range for applying such nitrogen-rich supplements in mushroom cultivation is typically between 750 and 1500 g per square meter of compost bed. The specific rates of 1000 and 1500 g/m² were chosen to evaluate the effect of two common and effective levels of these supplements on compost efficiency and subsequent mushroom yield.

Corresponding revision in the manuscript (Section 2.1.6): The following sentence has been added: "These specific application rates were selected based on the common commercial range for nitrogen-rich supplements (typically 750-1500 g/m²) to evaluate the effect of two effective and practically relevant levels on compost efficiency and mushroom yield."

2. Regarding the rationale for the specific percentages of recycled materials in the casing soil formulations:

The selected percentages were applied to the casing soil. The objective of creating this gradient was to find the optimal ratio that would maximize the utilization of recycled agricultural wastes in the casing layer while minimizing undesirable alterations in its physico-chemical properties compared to a standard commercial casing soil.

3. Regarding the basis for formulations C5 and C6:

All casing soil treatments, including C5 and C6, were formulated based on the gradient design logic explained in point 2, rather than on a prior optimization process. This experimental approach allows for a precise assessment of the incremental effects of increasing the proportion of recycled materials and helps identify the optimal balance between utilization efficiency and casing soil quality. The findings of this study will provide a scientific basis for designing optimized formulations in future research.

Corresponding revision in the manuscript (Section 2.1.3): The section now begins with the following clarification: "Seven distinct casing soil formulations were prepared and evaluated to determine an optimal ratio for incorporating recycled agricultural wastes. This gradient design was applied to all treatments and was not based on a prior optimization process. Rather, the experimental approach allowed for a precise assessment of the incremental effects of increasing the proportion of recycled materials, aiming to identify the optimal balance between utilization efficiency and the physico-chemical properties of the casing layer compared to standard commercial soils. The formulations were as follows:"

We believe these clarifications, now integrated into the manuscript, provide the necessary rationale for our experimental design choices.

**Comment 10:** Clarify how the bagasse fermentation time (6 months) was determined.

**Response:**
The six-month fermentation period for sugarcane bagasse was determined through pre-commercial trials. The primary goal was to reduce the high concentration of residual molasses (soluble carbohydrates) and lower the electrical conductivity (EC) to a suitable level for use as a cultivation substrate. The process involved approximately six cycles of washing and leaching over the six-month period. This extended timeframe allowed for the gradual and effective reduction of soluble sugars and salts, ultimately bringing the EC of the bagasse below the target threshold of 1500 µS/cm, which is considered acceptable for the intended application.

The added text in the manuscript in section 2.1.5 is as follows:

"Fresh bagasse was piled and initially leached for one week. Subsequently, the piles were turned and aerated monthly using a loader while being kept saturated with water. This fermentation and turning process was repeated over six months in approximately six cycles. The six-month period was determined through pre-commercial trials, with the primary goal of reducing the high concentration of residual molasses (soluble carbohydrates) and lowering the electrical conductivity (EC) to a level suitable for use as a cultivation substrate. This extended timeframe allowed for the gradual and effective reduction of soluble sugars and salts, ultimately bringing the EC of the bagasse below the target threshold of 1500 µS/cm, which is considered acceptable for the intended application. After this period, the bagasse was considered fully fermented and ready for use in the casing soil mixtures."

**Comment 11:** Explain why the recycled soil EC threshold (800–1500 µS/cm) was selected.
**Response:**
The electrical conductivity (EC) threshold of 800–1500 µS/cm for the recycled casing soil was selected based on established standards in mushroom cultivation, where mycelial growth is inhibited above approximately 2000 µS/cm due to osmotic stress. The upper limit of 1500 µS/cm provides a safe margin below this inhibitory level, while the lower limit of 800 µS/cm ensures sufficient nutrient availability. Accordingly, the six-month fermentation and leaching process for the bagasse was specifically designed to reduce the initial high EC and bring it into this optimal and safe range.

Reference:

Ouden, M. 2016. Mushroom Signal, A practical guide to optimal mushroom growing. Roodbont Publishers B.V., Zutphen, the Netherlands. 153 p.

The added text in the manuscript in section 2.1.4 is as follows:

"This specific threshold was selected based on established mushroom cultivation standards (Ouden, 2016). The upper limit of 1500 µS/cm provides a safe margin below the approximate inhibitory level of 2000 µS/cm, where higher EC can cause osmotic stress and inhibit mycelial growth, while the lower limit of 800 µS/cm ensures sufficient nutrient availability. "

**Comment 12:** The nutritional assessment uses ICP-OES and Kjeldahl, but No mention of calibration curves, standards, and instrument precision. Protein factor 4.38 is correct for mushrooms, but needs justification with a citation.

**Response:**

Thank you for the valuable comment. The methodological details for the instrumental analysis and the justification for the conversion factor are provided below.

1. ICP-OES Analysis for Mineral Elements:

The analysis was performed using a calibrated ICP-OES. The method was validated with the following parameters:

Calibration: Multi-point calibration curves were constructed daily using certified multi-element standard solutions.

Linear Range & Sensitivity: The established linear ranges, limits of detection (LOD), and limits of quantification (LOQ) for the reported elements are summarized in the table below.

Precision & Accuracy: The precision of the instrument was confirmed by repeated measurements of standards. The accuracy of the method for the specific matrix was verified through a recovery test using spiked samples, with results falling within the acceptable range of 94-104% for all reported elements.

ELEMENT Linear Range (µg/L) LOD (µg/L) LOQ (µg/L) Recovery (%)

Mg 10 - 5000 0.5 1.7 96 - 103

Mn 2 - 2000 0.2 0.7 95 - 102

Zn 5 - 3000 0.4 1.3 94 - 101

Cu 3 - 2500 0.3 1.0 95 - 104

2. Kjeldahl Method for Total Nitrogen:

The standard Kjeldahl method (digestion, distillation, and titration) was employed to determine total nitrogen.

Principle & Calibration: This is a primary volumetric titration method based on direct stoichiometry. The amount of nitrogen is calculated directly from the titer (normality) and volume of the standard sulfuric acid consumed. Therefore, it does not require a calibration curve.

Validation of Precision and Accuracy: The method's reliability was ensured by:

Analyzing all samples in triplicate.

Running method blanks in each batch.

Validating accuracy via the standard recovery method using Glycine as a certified reference material (CRM). The recovery rate was between 98-102%, confirming the method's accuracy for our sample matrix.

3. Nitrogen-to-Protein Conversion Factor:

The specific conversion factor of 4.38 (instead of the general factor of 6.25) was applied to convert the determined nitrogen content to crude protein. This factor is recommended for mushrooms due to their unique non-protein nitrogen content and amino acid profile, as per international food composition standards.

Justification Citation:

FAO/INFOODS. (2025). Guidelines for food composition data: Conversion factors for nitrogen to protein (Table 8). Food and Agriculture Organization of the United Nations.

The added text in the manuscript is presented in section 2.1.10 under the heading "Crude protein" and " Crude fiber".

**Comment 13:** The crude fiber method requires more detail (filter paper type, drying conditions, replicates).

**Response:**

The crude fiber analysis was conducted in triplicate (n=3) according to the standard gravimetric method, with the following specific experimental details incorporated. Briefly, 2 g of the dried and powdered mushroom sample underwent sequential reflux digestion with 150 mL of 0.255 N sulfuric acid followed by 150 mL of 0.313 N sodium hydroxide solution. After each digestion, the mixture was filtered through a Whatman No. 1 filter paper. The retained residue was thoroughly washed with distilled water. The filter paper containing the residue was then dried to a constant weight in an oven at 70°C for 48 hours, weighed, and subsequently ashed in a muffle furnace at 600°C for 4 hours. The crude fiber content was calculated based on the weight loss upon ashing, correcting for the ash content of the filter paper itself. The results are presented as the mean ± standard deviation of the three independent replicates.

The added text in the manuscript is presented in section 2.1.10 under the heading "Crude fiber ".

**Comment 14:** Mineral values vary widely between alternatives, possible sample variation is not discussed.

**Response:**

This is a valid and important point. We have acknowledged this limitation in the revised Discussion section. In the paragraph discussing the trade-off between yield and nutritional values, we have added the following sentence (paragraph 4):

"It is important to note that the considerable variation in mineral values between alternatives may also reflect inherent sample heterogeneity in raw materials or uneven mycelial distribution in the substrate, warranting cautious interpretation."

This addition provides a more balanced and critical perspective on the mineral data.
**Comment 15:** The GP model is good but under-explained. Weight selection is arbitrary (50–50–200). Why? The ranking approach before GP (Rank_pr, Rank_fe, Rank_ry) seems subjective. The target rank formula depends on ordinal data, not raw objective values. No model validation or robustness check. The authors are advised to justify all these in the GP model, provide a clear rationale for each weight scenario. Explain why ranking-based GP was chosen instead of classical weighted GP with objective scales.

**Response:**

We agree that further explanation and justification are necessary, and we have revised the manuscript accordingly to address each point raised.

1. Rationale for weight selection and scenarios: The reviewer correctly noted the initial arbitrary appearance of the weights. We have now clarified their origin. The weight scenarios (e.g., 2/3, 1/6, 1/6) were not chosen arbitrarily but were derived from a systematic sensitivity analysis. We tested a wide range of weight combinations and selected these specific sets because they represented distinct, practically meaningful decision-maker priorities (strong emphasis on a single objective vs. balanced objectives) and, crucially, because they produced significantly different ranking outcomes. This demonstrates that the model is sensitive to priority shifts and provides actionable insights for growers with different goals (e.g., profit-maximizing vs. nutrition-focused). This rationale has been added to Sections 2.2.2 and 3.2.4.

2. Justification for the ranking-based approach (Rank_pr, Rank_fe, Rank_ry): We acknowledge the concern regarding subjectivity. The ranking step serves as a normalization procedure to resolve the issue of incommensurable units. The raw objectives—profit (USD), nutritional index (dimensionless composite), and third-flush ratio (%)—have different scales and units, making direct weighted summation in a classical GP problematic. Converting raw values to ranks (1 to 35) places all three objectives on a common, dimensionless scale of relative performance. This method, known as preference ranking normalization, is established in Multi-Criteria Decision-Making (MCDM) for discrete alternative problems and effectively eliminates scale bias. We have added this justification to Section 2.2.2, citing relevant MCDM methodology literature.

3. Choice of ranking-based GP vs. classical weighted GP: As noted above, the primary reason is scale incompatibility. A classical weighted GP using raw values would implicitly give disproportionate influence to the objective with the largest numerical range (likely profit in USD). Our ranking-based GP focuses on the ordinal preference structure—how alternatives compare to each other on each objective—which is often more aligned with real-world decision-making ("which option is best overall?") than optimizing the sum of rescaled cardinal values. Furthermore, for a discrete choice problem among 35 alternatives, the final ranking is the direct output of interest to the decision-maker.

We agree that this is a critical component. The sensitivity analysis on the weights, presented in Table 3 and Fig. 7, serves as a primary robustness check. It shows how the optimal solution (top-ranked alternative) and the overall ranking stability change with varying preferences, validating that the model's outputs are logically consistent and interpretable across different scenarios.

Also, as per your comment 16, we have included a comprehensive validation step in the manuscript under the title "Model validation and robustness check".

**Comment 16:** Also, include a validation step, Sensitivity analysis beyond top-5 comparison and stability check using alternative weight sets. Further, I also suggest providing a worked numerical example for one alternative.

**Response:**

Thank you for your valuable suggestion regarding model validation and sensitivity analysis. We have incorporated a comprehensive validation step into the manuscript, which addresses your recommendation for an analysis beyond a simple top-5 comparison and includes a stability check using alternative weight sets.

1. Extended sensitivity and stability analysis (Monte Carlo simulation):

To rigorously assess the model's robustness against uncertainties in decision-maker preferences, we conducted a Monte Carlo simulation study (reported in a new subsection titled "Model validity assessment"). For each of the three priority scenarios (economic, food security, efficiency), we generated 100 random weight vectors under the constraints that the weights sum to 1 and the weight of the prioritized objective remains the highest. The ranking model was solved for each weight set, producing 100 alternative rankings per scenario. We analyzed the distribution of the mean rank difference for each alternative compared to its baseline rank (from the original deterministic weights).

2. Results and interpretation:

The results are visualized in scatter plots (new Figures 8, 9, and 10) and summarized statistically (new Table 5). The key findings are:

The mean rank differences across all alternatives and scenarios were relatively low (ranging from 0.98 to 3.65 on a scale of 1–35), indicating limited shifts in the overall ranking structure.

The Signal-to-Noise Ratio (SNR), calculated as the ratio of the mean rank difference to its standard deviation, exceeded 1 in all scenarios (1.75 to 2.97). According to quality engineering principles, this confirms that the ranking "signal" is substantially stronger than the "noise" induced by weight perturbations, attesting to high model stability and validity.

The clustering of points around the baseline in the figures visually confirms this stability.

**Comment 17:** Results and Discussion are descriptive but not analytical, and much of the discussion repeats the results without deeper insights.

**Response:**

We agree with the reviewer that the initial draft was overly descriptive. We have thoroughly revised the entire Discussion section to shift its focus from re-stating results to providing mechanistic explanations and analytical synthesis. Specifically, we have:

•Introduced concepts like "mechanistic synergy" and "physiological compromise" to frame the findings.

•Explicitly analyzed the "why" behind key observations, such as the superiority of the corn grit and casing C1 combination, moving beyond simply reporting the yield numbers.

•Dedicated a new paragraph to explaining the fundamental trade-off between yield and nutritional concentration through the established concepts of the dilution effect and natural bio fortification.

•Ensured that each result is now discussed in terms of its physiological, microbial, or practical implications, minimizing repetition and adding deeper scientific insight.

**Comment 18:** Also, add a mechanistic explanation of why corn grit performs better than soybean meal. How recycled casing alters water-retention, porosity, or microbial profile. How casing × supplement interactions influence flush distribution. Add mechanistic explanations supported by literature (e.g., nitrogen availability, microbial activation).

**Response:**

We have integrated the requested mechanistic explanations as follows:

1. Corn grit vs. Soybean meal: We now explain that corn grit acts as a "balanced, complex carbohydrate source" providing a "gradual nutrient boost" that sustains fructification without disrupting the substrate's balance. In contrast, soybean meal is a "rapid-release nitrogen supplement" whose negative effects are mechanistically detailed via ammonia toxicity, promotion of vegetative growth, and stimulation of competitive microbial populations (citing Mami et al., 2013; Burton & Noble, 2015; Tello Martín et al., 2022).

2. Recycled casing (SMS - Spent Mushroom Substrate): We have incorporated the suggested point. In paragraph 2, we state that SMS-based casings (like C5), "while potentially altering microbial profiles and water retention (Yücel, 2025), cannot compensate for a deliberate and balanced nutritional input." This connects the physical/microbial alteration to the observed yield outcome.

3. Casing × Supplement interaction on flush distribution: We have added a sentence proposing this mechanism: "Furthermore, the casing × supplement interaction likely influences flush dynamics; optimal pairings like C1S4 may support sustained mycelial health across flushes, whereas incompatible ones like C4S3 could lead to a rapid decline after the first flush." This provides a testable hypothesis for future, more detailed studies on flush-by-flush data.

**Comment 19:** Also, discuss implications for the circular economy and peat reduction more critically. Explain why some high-yield treatments show poorer nutritional values.

**Response:**

In response to your comment, a more critical discussion about environmental consequences and the circular economy was added (paragraph 5).

We have addressed this central question by adding a dedicated analytical paragraph (paragraph 6) that introduces and applies the key concepts of the dilution effect and biofortification.

We explain that high-yield treatments prioritize biomass and water accumulation, leading to a dilution of minerals and proteins per unit weight (citing Zicari et al., 2012; Pardo-Giménez et al., 2012).

Conversely, we clarify that lower-yield, high-nutrient treatments (e.g., C1S3) result from natural biofortification, where nitrogen-rich supplements stimulate mycelial metabolism to enhance uptake and translocation of elements like Cu and Zn, producing denser, nutrient-concentrated tissues (citing Atila, 2017; Rzymski et al., 2017).

This explanation moves beyond mere correlation to establish a clear physiological cause-and-effect relationship for the observed inverse pattern, which is a critical insight for guiding cultivation strategies for different market goals.

**Comment 20:** In my opinion, the conclusions overstate findings, the conclusion claims the model provides a “powerful and adaptable tool,” but no real-world testing or economic validation was presented. Justify?

**Response:**
We agree with the reviewer that the original claim required stronger, quantitative validation. To address this directly and substantiate the model's robustness, we have conducted a Monte Carlo simulation-based validation. In light of this new evidence, we have tempered the language in the conclusion. The phrase powerful and adaptable tool" is now justified by reference to the Monte Carlo stability.

**Comment 21:** Many references are outdated or unrelated to specific claims.

**Response:**

We thank the reviewer for this essential feedback on our literature review. We have conducted a thorough revision of the reference list to ensure it is current, relevant, and directly supportive of our specific claims.

**Comment 22:** Some references are unpublished (“Sugarcane & Byproduct Company 2024”).

**Response:**

Due to the removal of text from the introduction, this reference is no longer in the text.

**Comment 23:** Check consistency in citation styles.

**Response:**

We have conducted a thorough, line-by-line check of all in-text citations and the corresponding reference list to ensure full and consistent adherence to the required citation style.

**Reviewer #3:**

The paper is very long, contains methodological ambiguities, statistical clarity issues, weak justification for modeling decisions, and several writing and formatting problems that require attention before acceptance.

**Response:**

We sincerely thank Reviewer #3 for their time and comprehensive assessment of our manuscript. We acknowledge the concerns raised regarding the paper's length, methodological clarity, statistical reporting, justification of modeling choices, and writing quality. We have undertaken a substantial revision to address each of these points comprehensively.

**1. Length and structure:** We have carefully edited the manuscript to improve conciseness. Redundant descriptions and lengthy introductory passages have been removed or condensed, and the flow between sections has been streamlined. The revised version presents a more focused and efficient narrative without compromising essential scientific content.

**2. Methodological and statistical clarity:**

- A complete ANOVA table with all p-values and a clear definition of main-plot and sub-plot errors has been added to the results.
- All relevant figures now include error bars (SE), statistical grouping letters with clear explanations, explicit sample sizes, and fully labeled axes (including units or the nature of indices).
- The statistical analysis section (Section 2.1) now explicitly states the sample size for all measurements, confirms that data met ANOVA assumptions without transformation, and specifies the post-hoc test used.

**3. Justification for modeling decisions:** The manuscript has been revised to better articulate the rationale behind key modeling choices:

- The choice of Goal Programming (GP) over other optimization methods is now explicitly justified in terms of its suitability for handling multiple, often conflicting, objectives in agricultural decision-making.
- The selection and weighting of the three specific objectives (economic profit, nutritional index, third-flush yield ratio) are now more clearly linked to documented grower priorities and industry challenges.
- The robustness of the GP model is now demonstrated through an extended sensitivity analysis presented in a new figures/table, showing how variations in objective weights affect the optimal solution.

**4. Writing and formatting:**

- The manuscript has been thoroughly proofread to correct grammatical errors, improve sentence structure, and ensure consistent formatting.
- Informal or narrative language (including the overuse of apostrophe *'s*) has been replaced with precise, technical prose.
- References have been checked for consistency in style, and several have been updated to more current and directly relevant sources.

We believe these revisions have directly addressed the reviewer's valid criticisms. The manuscript is now more concise, methodologically transparent, statistically rigorous, and clearly written. We are confident that these improvements meet the standards required for publication and thank the reviewer again for the constructive feedback that has strengthened our work.

At the end, thanks to the respected reviewers who spent a lot of time and energy scrutinizing this manuscript, please, if the explanations and corrections made are not enough, they will express their views on the need to take action to investigate and enforce them.

All the best,

Corresponding author, also on behalf of my co-authors
