# Supplementary material for: Economic, nutritional, and efficiency assessment of recycled casing soil and compost nutritional supplements for sustainable white button mushroom cultivation
Source: PLoS One. 2026 Aug 13;21(8):e0337146. doi: 10.1371/journal.pone.0337146 (PMC13472422; doi:10.1371/journal.pone.0337146)
Supplement: Supplementary file 10 [file pone.0337146.s006.docx]

**Dear Editors and Reviewers,**

We hope this message finds you well. We are pleased to submit the revised version of our manuscript entitled **“Economic, Nutritional, and Efficiency Assessment of Recycled Casing Soil and Compost Nutritional Supplements for Sustainable White Button Mushroom Cultivation.”**

We have carefully considered all the reviewers' comments and have revised the manuscript accordingly. For clarity, each reviewer's comments have been addressed in the following color-coded sections:

- **Reviewer 2:** Pink highlights
- **Reviewer 3:** Green highlights

We believe that the revisions have significantly strengthened the manuscript, and we are grateful for the insightful and constructive feedback provided by the reviewers.

Thank you for your time and consideration.

**Reviewer #2:**

The authors have addressed most of the reviewers comments. In my opinion, the manuscript still reads more like an applied agronomic study than a mechanistically grounded contribution to food/biochemical or bioprocess science, which may remain a concern for editorial acceptance.

**Comment 1:** The abstract now mentions “metabolic and enzymatic pathways”, but no specific enzymes, transporters or biosynthetic routes were included. The claims remain largely conceptual and not supported by experimental validation.

**Response:** We truly appreciate the reviewer’s time and careful reading of our manuscript. That said, we’d like to gently point out that the phrase “metabolic and enzymatic pathways” never actually appears in the abstract of any version we submitted. Our abstract—and really the whole manuscript—focuses on economic profitability, nutritional quality, and production efficiency, all within a goal‑programming framework for sustainable mushroom cultivation. The main contribution of our work is a multi‑objective model that combines experimental agronomic data with economic and nutritional indicators, not a mechanistic deep dive into molecular pathways.

Still, to address the reviewer’s concern about mechanistic depth, we’ve added brief discussions of relevant enzymes (like superoxide dismutase, ATPases, and tyrosinase) and transporters (such as the ZIP family, MgtE, and NRAMP) in both the Introduction and Discussion sections—these changes are detailed in our responses to comments #2 and #3. We hope these clarifications and additions strike the right balance and satisfy the reviewer’s concern, while keeping the focus where it belongs: on the applied agronomic and economic nature of our study.

**Comment 2:** No dose–response framework or physiological threshold discussion is included. Specify why specific mineral precursors were selected or whether they act as enzyme cofactors, transcriptional regulators, or structural elements.

**Response:** We appreciate the reviewer raising this important point. To address it, we’ve made substantial revisions to the manuscript, adding the requested dose–response framework, physiological thresholds, and a clear rationale for selecting Mg, Zn, Cu, and Mn. Here’s a summary of the key changes.

In the Introduction (new paragraph after line 5), we now explicitly explain why we chose these four minerals. Specifically, we discuss their well‑established physiological roles in Agaricus bisporus—as enzyme cofactors (e.g., Zn in superoxide dismutase, Mg in ATPases, Cu in cytochrome c oxidase, Mn in Mn‑SOD), as regulators (Zn‑finger proteins), and as structural components (Mg in ribosomes). This directly responds to the reviewer’s request to clarify the mode of action of these mineral precursors.

In the Results (section 3.2.2, Nutritional characteristics analysis), we’ve added the dose‑response analysis. We now describe that increasing soybean meal from 1000 to 1500 g m⁻² led to a substantial rise in the Food Elements Index (FEI), reflecting a clear linear dose‑dependent pattern. In contrast, corn grit showed only a marginal increase beyond 1000 g m⁻², suggesting transport saturation already at the lower dose. These observations are presented without over‑specifying numerical values beyond our experimental resolution.

In the Discussion, we inserted a new mechanistic paragraph right after the sentence beginning “The observed inverse relationship between high yield and superior nutritional values…”. There we explain the physiological thresholds for nitrogen (soybean meal) and carbohydrate (corn grit) supplementation. For soybean meal, we note that 1500 g m⁻² approaches the nitrogen tolerance threshold; above that, ammonia‑mediated stress would likely compromise yield. For corn grit, the near‑saturation of mineral uptake at 1000 g m⁻² points to a finite transport capacity for divalent cations, probably mediated by ZIP‑family transporters. This paragraph also revisits the cofactor and structural roles of the selected minerals, linking the dose‑response patterns directly to metabolic saturation.

Together, these revisions provide the previously missing dose‑response framework, clarify the physiological basis for our mineral selection, and address all three mechanistic categories requested by the reviewer (enzyme cofactors, transcriptional regulators, and structural elements). We believe the manuscript is now much stronger in this respect.

**Comment 3:** increase in Fe/Zn/Mg is reported but still no discussion on, competitive ion uptake, transport saturation or potential antagonistic effects between minerals.

**Response:** We’ve gone back and substantially revised the Discussion to better address competitive uptake, transport saturation, and antagonistic effects—specifically for the cations we actually measured: Mg, Zn, Cu, and Mn. The reviewer mentioned iron, but to be clear, Fe wasn't part of our mineral panel. Our rationale, which we've already laid out in the Introduction, focuses on Mg, Zn, Cu, and Mn because of their established roles in human health. That said, we didn't ignore the mechanistic side. The revised Discussion now covers three key points: (i) the shared ZIP‑family transporters for Zn²⁺ and Cu²⁺ and the resulting potential for competition; (ii) clear evidence of transport saturation, as seen in the plateau effect with corn grit; and (iii) antagonistic interactions—specifically between Ca²⁺ (from the CaCO₃ in the casing layer) and Mg²⁺, as well as between Zn²⁺ and Cu²⁺.

**Comment 4:** Tables again lack sample size (n).

**Response:** We thank the reviewer for this careful observation.

In Table 3 (ANOVA table), the degrees of freedom (total df = 104, error df = 56) reflect the underlying sample size of 105 experimental units (35 treatments × 3 replications). However, to make this explicit for readers, we have added a footnote to Table 3 stating: "The total number of experimental units was 105 (35 treatments × 3 replicates)."

We have now added an explicit statement of sample size to all relevant tables and figures. Specifically, we added the following note to Tables 3, 4, and Appendix A3, as well as to Figures 3, 4, 5, and 6:

"Note: Each treatment combination was replicated three times (n = 3 per treatment)"

This note now appears alongside each table and figure where experimental data are presented. We believe this addresses the reviewer's concern completely. Additionally, the sample size (n = 3) was already explicitly stated in the Materials and Methods section (sections 2.1.2 and 2.1.10).

Thank you again for the valuable feedback.

**Comment 5:** Figures do not include, error bar definition (SD vs. SE)

**Response:** We thank the reviewer for this observation. We have added error bars to all figures (Figures 3, 4, 5, and 6) and have clearly defined the type of error bar used.

Error bars represent Standard Error of the Mean (SE), not Standard Deviation (SD). We chose SE because it is the appropriate measure for showing the precision of the sample mean when comparing treatment effects.

A note stating "Error bars represent the standard error of the mean (±SE) calculated from the raw data" has been added beneath each figure (Figures 3, 4, 5, and 6).

The definition of SE (SE = SD / √n) and the sample size (n = 3) are explicitly stated in the Materials and Methods section (sections 2.1.2 and 2.1.10).

**Comment 6:** Ethical, safety, and regulatory context still missing, Food safety thresholds for mineral fortification, regulatory limits (FSSAI/WHO/EFSA), add discussion on these aspects.

**Response:** Thanks to comment #6, we've now added a dedicated paragraph in the Discussion—right before the Conclusion—that directly addresses food safety. Specifically, we reference the relevant guidelines from EFSA, WHO/FAO, and FSSAI for Zn, Cu, and Mg, convert our dry‑weight concentrations into estimated intake per serving on a fresh‑weight basis, and confirm that all values stay within the tolerable upper intake levels. We also note that there are currently no regulatory limits specifically for these minerals in edible mushrooms. The paragraph includes a brief ethical note (no approval required) and a practical recommendation: periodic heavy metal monitoring when recycled casing soil is being reused. This addition fills the regulatory and safety gap the reviewer pointed out.

**Reviewer #3:**

**Comment 1:** The introduction is unusually long, overly descriptive, and contains repeated justifications.

**Response:** We thank the reviewer for this observation. We have thoroughly revised the Introduction to make it more concise while preserving all original references and the core logical structure. The revised version is approximately 30-35% shorter. Specifically, we:

- Removed redundant justifications (e.g., repeated explanations of mushroom benefits and peat extraction problems that appeared in multiple paragraphs).
- Condensed descriptive passages without removing any citations.
- Streamlined the paragraph on goal programming by eliminating repetitive explanations of its philosophy.
- Restructured the novelty paragraph to be more direct and less verbose.

The revised Introduction is now more focused and reader-friendly while retaining all original references and technical content.

**Comment 2:**	Why not use multi-criteria methods like: TOPSIS, AHP, MOORA, Multi-objective linear programming directly (without ranking)?

**Response:** We thank the reviewer for this insightful methodological question. The choice of goal programming (GP) over other multi-criteria methods was deliberate and is justified by the specific nature of our problem, as explained below.

1. The nature of our objectives: targets, not just rankings

Methods such as TOPSIS, AHP, and MOORA are primarily designed to rank alternatives based on their relative closeness to an ideal solution or pairwise comparisons. However, our decision problem involves three conflicting objectives for which we have specific target values derived from experimental data. Goal programming is uniquely suited for such problems because it minimizes deviations from predefined targets (whether shortfall or excess), rather than merely producing a relative ranking. This target-based approach aligns better with real-world decision-making, where growers have aspirational goals (e.g., achieving a certain profit level or limiting third-flush yield to below 10%) rather than simply seeking the "best" among imperfect options.

2. The discrete nature of our alternatives

Multi-objective linear programming (direct optimization without ranking) typically assumes continuous decision variables (e.g., how much of each ingredient to mix). In our study, however, the alternatives are discrete – they arise from specific, predefined combinations of casing formulations (C1 to C7) and supplement levels (S1 to S5) tested in the field experiment. These 35 discrete alternatives cannot be represented as continuous variables. Therefore, a discrete multi-criteria decision-making (MCDM) approach, such as GP with binary variables, is methodologically appropriate, whereas continuous multi-objective LP is not feasible for this problem structure.

3. Robustness through sensitivity analysis

While AHP is powerful for deriving weights from pairwise comparisons, it assumes that the decision-maker can consistently compare all pairs of criteria – a demanding task when three objectives are incommensurable. Our GP model, in contrast, allows direct specification of weights (based on producer priorities) and includes a comprehensive Monte Carlo simulation (100 random weight vectors per scenario) to test the robustness of rankings. This level of sensitivity analysis is less common in standard TOPSIS or MOORA applications.

4. Alignment with real-world producer priorities

In practice, mushroom growers do not think in terms of "which alternative is closest to an ideal point." Instead, they ask: "Given my profit target, my desired nutritional quality, and my willingness to accept a longer growing cycle, which combination minimizes my disappointment across all goals?" This is precisely what GP does: it minimizes the weighted sum of deviations from targets. The ability to assign different weights to positive and negative deviations (over-achievement vs. under-achievement) is another strength of GP not shared by TOPSIS or MOORA.

For the reasons above – target-based deviation minimization, discrete alternative structure, robustness via sensitivity analysis, and alignment with real-world decision logic – goal programming was the most appropriate method for our study. We thank the reviewer again for the opportunity to clarify this important point.

**Comment 3:** The figures need Improvement as many graphs lack error bars and Axis labels are not visible or too small.

**Response:** We thank the reviewer for this observation. Error bars have been added to all figures (Figures 3, 4, 5, and 6). A clear definition has been provided beneath each figure stating: "Error bars represent the standard error of the mean (±SE) calculated from the raw data." We used SE (not SD) as it is the appropriate measure for the precision of the sample mean. We have also increased the font size of all axis labels to ensure they are clearly visible. All figures have been regenerated with higher resolution. The revised figures now meet the journal's standards.

**Comment 4:** The result section reads like a raw extraction from analyses. More synthesis is needed.

**Response:** The reviewer is correct. We have substantially rewritten the Results section to move beyond mere description of numbers and to provide meaningful synthesis. Specifically, we made the following revisions:

- We began each subsection with a topic sentence that states the main pattern or finding.
- We added interpretive statements that explain why a pattern matters (for example, we explained that the 47% yield difference between supplements indicates supplement choice is more critical than casing type).
- We explicitly highlighted trade-offs, such as the inverse relationship between high-yield and high-nutrition formulations.
- We drew practical implications where appropriate, including that corn grit at 1500 g/m² allows early termination of the third flush with associated energy and cost savings.

All original data, numbers, statistical results, and references remain unchanged. Only the presentation has been enhanced to provide synthesis and interpretation. These revisions have been completed throughout section 3.

**Comment 5:** Several sentences are very long, contain redundancies, or use overly formal/unnecessary phrasing.

**Response:** We have carefully edited the entire manuscript to improve sentence fluency. Specific changes include:

Breaking overly long sentences into shorter, clearer ones.

Removing redundant phrases such as "in other words," "thereby," "constitutes the only," and "concurrently."

Replacing overly formal constructions (e.g., "can be attributed to" → "stems from"; "the findings clearly demonstrate" → no unnecessary adverb).

Eliminating unnecessary adjectives and adverbs without changing meaning.

**Comment 6:**	Rewrite your Abstract

**Response:** The Abstract has been completely rewritten. The revised version is more concise, result-oriented, and follows a clear structure: problem statement, methods, key results, and conclusion. Unnecessary descriptive phrases have been removed, and the main quantitative finding (optimal combination C5+S5) is now stated upfront.

**Comment 7:** Improve the discussion

**Response:** The Discussion has been substantially improved. We have:

- Restructured it into five clear thematic paragraphs: (1) summary of key findings, (2) physiological mechanisms underlying the trade-offs, (3) comparison with existing literature, (4) circular economy and peat reduction implications, and (5) limitations and future research.
- Added interpretive synthesis rather than simply repeating results.
- Removed redundant explanations and overly long sentences.
- Strengthened the connection between findings and practical applications for growers.

The revised Discussion now provides a more coherent, insightful, and useful interpretation of the findings for both researchers and practitioners.

At the end, thanks to the respected reviewers who spent a lot of time and energy scrutinizing this manuscript, please, if the explanations and corrections made are not enough, they will express their views on the need to take action to investigate and enforce them.

All the best,

Corresponding author, also on behalf of my co-authors
