# Supplementary material for: Economic, nutritional, and efficiency assessment of recycled casing soil and compost nutritional supplements for sustainable white button mushroom cultivation
Source: PLoS One. 2026 Aug 13;21(8):e0337146. doi: 10.1371/journal.pone.0337146 (PMC13472422; doi:10.1371/journal.pone.0337146)
Supplement: Peer review history

# Peer review: Decision Letter 0

**DOI:** 10.1371/journal.pone.0337146.r001

**Authors:** Nishant Kumar

24 Nov 2025

Dear Dr. Kalateh Jari,

Thank you for submitting your manuscript to PLOS ONE. After careful consideration, we feel that it has merit but does not fully meet PLOS ONE’s publication criteria as it currently stands. Therefore, we invite you to submit a revised version of the manuscript that addresses the points raised during the review process.

Please submit your revised manuscript by Jan 08 2026 11:59PM. If you will need more time than this to complete your revisions, please reply to this message or contact the journal office at plosone@plos.org. When you're ready to submit your revision, log on to https://www.editorialmanager.com/pone/ and select the 'Submissions Needing Revision' folder to locate your manuscript file.

- A rebuttal letter that responds to each point raised by the academic editor and reviewer(s). You should upload this letter as a separate file labeled 'Response to Reviewers'.
- A marked-up copy of your manuscript that highlights changes made to the original version. You should upload this as a separate file labeled 'Revised Manuscript with Track Changes'.
- An unmarked version of your revised paper without tracked changes. You should upload this as a separate file labeled 'Manuscript'.

If you would like to make changes to your financial disclosure, please include your updated statement in your cover letter. Guidelines for resubmitting your figure files are available below the reviewer comments at the end of this letter.

If applicable, we recommend that you deposit your laboratory protocols in protocols.io to enhance the reproducibility of your results. Protocols.io assigns your protocol its own identifier (DOI) so that it can be cited independently in the future. For instructions see: https://journals.plos.org/plosone/s/submission-guidelines#loc-laboratory-protocols. Additionally, PLOS ONE offers an option for publishing peer-reviewed Lab Protocol articles, which describe protocols hosted on protocols.io. Read more information on sharing protocols at https://plos.org/protocols?utm_medium=editorial-email&utm_source=authorletters&utm_campaign=protocols.

We look forward to receiving your revised manuscript.

Kind regards,

Nishant Kumar, Ph.D

Academic Editor

PLOS ONE

Journal requirements:

When submitting your revision, we need you to address these additional requirements.

1. Please ensure that your manuscript meets PLOS ONE's style requirements, including those for file naming. The PLOS ONE style templates can be found at

https://journals.plos.org/plosone/s/file?id=wjVg/PLOSOne_formatting_sample_main_body.pdf and

https://journals.plos.org/plosone/s/file?id=ba62/PLOSOne_formatting_sample_title_authors_affiliations.pdf

2. PLOS requires an ORCID iD for the corresponding author in Editorial Manager on papers submitted after December 6th, 2016. Please ensure that you have an ORCID iD and that it is validated in Editorial Manager. To do this, go to ‘Update my Information’ (in the upper left-hand corner of the main menu), and click on the Fetch/Validate link next to the ORCID field. This will take you to the ORCID site and allow you to create a new iD or authenticate a pre-existing iD in Editorial Manager.

3. Please amend your authorship list in your manuscript file to include author Sepideh Kalateh Jari, Ali Eslamizadeh, Ali Mohammadi Torkashvand, Mohammad Farsi, Mohamadreza Salehi Salmi.

4. Please amend your list of authors on the manuscript to ensure that each author is linked to an affiliation. Authors’ affiliations should reflect the institution where the work was done (if authors moved subsequently, you can also list the new affiliation stating “current affiliation:….” as necessary).

5. If the reviewer comments include a recommendation to cite specific previously published works, please review and evaluate these publications to determine whether they are relevant and should be cited. There is no requirement to cite these works unless the editor has indicated otherwise.

Additional Editor Comments (if provided):

[Note: HTML markup is below. Please do not edit.]

Reviewers' comments:

Reviewer's Responses to Questions

**Comments to the Author**

1. Is the manuscript technically sound, and do the data support the conclusions?

Reviewer #1: Yes

Reviewer #2: Partly

Reviewer #3: Partly

**********

2. Has the statistical analysis been performed appropriately and rigorously?

Reviewer #1: Yes

Reviewer #2: No

Reviewer #3: No

**********

3. Have the authors made all data underlying the findings in their manuscript fully available?

The PLOS Data policy

Reviewer #1: Yes

Reviewer #2: No

Reviewer #3: Yes

**********

4. Is the manuscript presented in an intelligible fashion and written in standard English?

Reviewer #1: No

Reviewer #2: No

Reviewer #3: Yes

**********

Reviewer #1: The manuscript presents a well-designed and methodologically sound study addressing sustainable cultivation strategies for Agaricus bisporus using a goal programming (GP) framework. The integration of field experiments with optimization modeling is commendable and provides both scientific and practical insights into sustainable mushroom production systems. The topic is timely and aligns with global sustainability and circular economy goals. However, several minor issues related to structure, clarity, and presentation should be addressed to enhance readability and overall impact.

1. The abstract and conclusion should be revised to provide a technical glimpse of overall study.

2. Throughout the manuscript, there is extensive use of apostrophe s ('s) which pertains to a story telling method rather than a technically proficient language.

Reviewer #2: I had critically gone through the MS and observed the following:

The concept of MS is good, and the combination of experimental and decision-optimization approaches is the need of the hour in mushroom cultivation. However, the manuscript requires substantial revisions before it can be considered for publication.

1. The introduction contains an excessive amount of general information about mushrooms, sustainability, and the sugarcane sector in Iran. The objectives of the study are missing, and the authors are advised to include a paragraph explaining how the present study is unique and novel compared to existing ones. Focus more sharply on the knowledge gap:

o How casing soil and supplement interactions are not yet optimized under multi-objective settings.

o Why is integrating agronomic data with GP modeling scientifically justified?

2. Move regional sugarcane industry details to the Discussion or as a brief contextual note.

3. The manuscript mentions ANOVA and Tukey’s test, but key elements are missing, including Mean ± SE values in figures or tables, whether data were transformed (especially percentage and ratio data), and the Number of samples for nutritional analysis.

4. Authors are suggested to provide a full ANOVA table with all p-values and also clearly define the Main plot error and the Subplot error.

5. Add sample size (n) for all parameters, including mineral analysis.

6. Add measures of variation (SE/SD) to Fig. 3, 5, and 6.

7. Figures lack clarity and essential information. Figures 3–6 do not include: Error bars, Sample sizes, Statistical groupings (only letters without explanation), Units on axes for nutritional index and ratios. Rectify these mistakes.

8. The manuscript does not explain:

• Why 1000 and 1500 g/m² were chosen for soybean/corn.

• Why do recycled soil fractions follow exactly the chosen percentages?

• Whether C5 and C6 formulations were based on previous optimization trials.

9. The authors are advised to cite prior work or provide preliminary results supporting chosen levels.

10. Clarify how the bagasse fermentation time (6 months) was determined.

11. Explain why the recycled soil EC threshold (800–1500 µS/cm) was selected.

12. The nutritional assessment uses ICP-OES and Kjeldahl, but No mention of calibration curves, standards, instrument precision. Protein factor 4.38 is correct for mushrooms, but needs justification with a citation.

13. The crude fiber method requires more detail (filter paper type, drying conditions, replicates).

14. Mineral values vary widely between alternatives, possible sample variation is not discussed.

15. The GP model is good but under-explained. Weight selection is arbitrary (50–50–200). Why? The ranking approach before GP (Rank_pr, Rank_fe, Rank_ry) seems subjective. The target rank formula depends on ordinal data, not raw objective values. No model validation or robustness check. The authors are advised to justify all these in the GP model, provide a clear rationale for each weight scenario. Explain why ranking-based GP was chosen instead of classical weighted GP with objective scales.

16. Also, include a validation step, Sensitivity analysis beyond top-5 comparison and stability check using alternative weight sets. Further, I also suggest providing a worked numerical example for one alternative.

17. Results and Discussion are descriptive but not analytical, and much of the discussion repeats the results without deeper insights.

18. Also, add a mechanistic explanation of why corn grit performs better than soybean meal. How recycled casing alters water-retention, porosity, or microbial profile. How casing × supplement interactions influence flush distribution. Add mechanistic explanations supported by literature (e.g., nitrogen availability, microbial activation).

19. Also, discuss implications for the circular economy and peat reduction more critically. Explain why some high-yield treatments show poorer nutritional values.

20. In my opinion, the conclusions overstate findings, the conclusion claims the model provides a “powerful and adaptable tool,” but no real-world testing or economic validation was presented. Justify?

21. Many references are outdated or unrelated to specific claims.

22. Some references are unpublished (“Sugarcane & Byproduct Company 2024”).

23. Check consistency in citation styles.

Recommendation: Major revisions are required before its acceptance.

Reviewer #3: the paper is very long, contains methodological ambiguities, statistical clarity issues, weak justification for modeling decisions, and several writing and formatting problems that require attention before acceptance.

**********

what does this mean?). If published, this will include your full peer review and any attached files.

If you choose “no”, your identity will remain anonymous but your review may still be made public.

**Do you want your identity to be public for this peer review?**  For information about this choice, including consent withdrawal, please see our Privacy Policy

Reviewer #1: No

Reviewer #2: **Yes:**  Aditya

Reviewer #3: No

**********

[NOTE: If reviewer comments were submitted as an attachment file, they will be attached to this email and accessible via the submission site. Please log into your account, locate the manuscript record, and check for the action link "View Attachments". If this link does not appear, there are no attachment files.]

To ensure your figures meet our technical requirements, please review our figure guidelines: https://journals.plos.org/plosone/s/figures

You may also use PLOS’s free figure tool, NAAS, to help you prepare publication quality figures: https://journals.plos.org/plosone/s/figures#loc-tools-for-figure-preparation.

NAAS will assess whether your figures meet our technical requirements by comparing each figure against our figure specifications.

Attachment Submitted filename: Reviewer comments.docx

# Author comment: Author response to Decision Letter 1

**DOI:** 10.1371/journal.pone.0337146.r002

8 Jan 2026

Dear Editors and Reviewers,

We hope this message finds you well. We are pleased to submit the revised version of our manuscript entitled “Economic, Nutritional, and Efficiency Assessment of Recycled Casing Soil and Compost Nutritional Supplements for Sustainable White Button Mushroom Cultivation.”

We have carefully considered all the reviewers' comments and have revised the manuscript accordingly. For clarity, each reviewer's comments have been addressed in the following color-coded sections:

• Reviewer 1: Yellow highlights

• Reviewer 2: Green highlights

• Reviewer 3: Yellow & Green highlights

We believe that the revisions have significantly strengthened the manuscript, and we are grateful for the insightful and constructive feedback provided by the reviewers.

Thank you for your time and consideration.

Reviewer #1:

The manuscript presents a well-designed and methodologically sound study addressing sustainable cultivation strategies for Agaricus bisporus using a goal programming (GP) framework. The integration of field experiments with optimization modeling is commendable and provides both scientific and practical insights into sustainable mushroom production systems. The topic is timely and aligns with global sustainability and circular economy goals. However, several minor issues related to structure, clarity, and presentation should be addressed to enhance readability and overall impact.

1. The abstract and conclusion should be revised to provide a technical glimpse of overall study.

2. Throughout the manuscript, there is extensive use of apostrophe s ('s) which pertains to a story telling method rather than a technically proficient language.

Response:

We sincerely thank Reviewer #1 for their positive assessment of the study's design, methodology, and relevance to sustainability goals. We are pleased that the integration of field experiments with optimization modeling was found to be commendable.

We have carefully addressed the two specific points raised to enhance the manuscript's technical proficiency and clarity:

1. Revision of abstract and conclusion: As suggested, we have thoroughly revised both the Abstract and the Conclusion sections. The revisions ensure these sections provide a clear, concise, and technically precise overview of the study's objectives, methodology, key findings, and implications, moving from a descriptive to a more analytical summary.

2. Correction of language style: We acknowledge the reviewer's point regarding the informal use of the apostrophe 's (possessive/genitive case) in a narrative style. We have performed a comprehensive edit throughout the entire manuscript to replace this colloquial construction with more formal and precise technical language. For instance, phrases like "the study's goal" have been revised to "the goal of this study" or "the study objective." This revision ensures the manuscript adheres to the standard, objective prose expected in scientific literature.

We believe these revisions have strengthened the manuscript's structure, clarity, and professional tone. We thank the reviewer again for their constructive feedback.

Reviewer #2:

I had critically gone through the MS and observed the following:

The concept of MS is good, and the combination of experimental and decision-optimization approaches is the need of the hour in mushroom cultivation. However, the manuscript requires substantial revisions before it can be considered for publication.

Comment 1: The introduction contains an excessive amount of general information about mushrooms, sustainability, and the sugarcane sector in Iran. The objectives of the study are missing, and the authors are advised to include a paragraph explaining how the present study is unique and novel compared to existing ones. Focus more sharply on the knowledge gap.

Response:

Thank you for your insightful feedback regarding the Introduction section. We have carefully revised it to address your concerns by focusing more sharply on the knowledge gap, clarifying the novelty of our study, and explicitly stating the research objectives. The key changes are as follows:

The paragraph on the sugarcane industry in Iran has been removed, and general background information on mushrooms has been condensed to maintain a sharper focus on the research problem.

A new paragraph has been added towards the end of the Introduction to clearly state the research gap: the lack of an integrated framework for simultaneously optimizing the interactions between recycled casing soils and nutritional supplements under multiple objectives (economic, nutritional, and efficiency), and for merging empirical agronomic data with multi-criteria decision modeling. This paragraph explicitly presents our two-phase experimental-modeling approach as the novel contribution addressing this gap.

Immediately following the novelty statement, a dedicated paragraph now lists the three specific research objectives in a numbered format:

Objective 1: To evaluate the effects of casing soils and supplements via field experiments.

Objective 2: To develop a Goal Programming model for simultaneous optimization of the three competing goals.

Objective 3: To identify the optimal strategy through sensitivity analysis and provide a practical decision-support tool.

These revisions ensure that the scientific justification, novelty compared to prior work, and clear research aims are now prominently and concisely highlighted in the Introduction.

Comment 1.1: How casing soil and supplement interactions are not yet optimized under multi-objective settings.

Response:

Thank you for your insightful comment regarding the optimization of casing soil and supplement interactions under multi-objective settings.

In this study, we explicitly modeled and optimized the interaction between casing soil formulations and nutritional supplements within a multi-objective Goal Programming (GP) framework. Each of the 35 treatment combinations (7 casing soils × 5 supplements) was evaluated as an independent alternative and ranked based on three objectives: economic profit, nutritional index, and third-flush yield ratio.

Through the GP model, we not only ranked all alternatives but also conducted sensitivity analysis (Table 3 and Fig. 7) to demonstrate how different priority weightings (economic, nutritional, efficiency) lead to distinct optimal combinations of casing soil and supplement. For example:

When economic profit is prioritized, the optimal combination is C5 (20% Tima peat + 40% recycled soil + bagasse) with S4 (corn grit at 1000 g/m²).

When nutritional quality is emphasized, C2 (95% Tima peat) with S5 (corn grit at 1500 g/m²) performs best.

When efficiency is prioritized, C5 with S5 (corn grit at 1500 g/m²) is selected.

Thus, the interaction between casing soil and supplement is systematically optimized under multi-objective preferences, and the model provides a decision-support tool for selecting the best combination based on grower priorities.

Comment 1.2: Why is integrating agronomic data with GP modeling scientifically justified?

Response:

The integration of agronomic experimental data with Goal Programming (GP) modeling is scientifically justified for the following reasons:

1. Bridging the gap between agronomic research and practical decision-making: While field experiments provide valuable data on crop performance under different treatments, they do not inherently resolve trade-offs between conflicting objectives (e.g., profit vs. nutrition vs. sustainability). GP provides a structured framework to incorporate these experimental results into a multi-objective decision model, enabling the identification of optimal strategies that balance competing priorities.

2. Handling multi-dimensional agricultural challenges: Sustainable agriculture inherently involves multiple and often conflicting goals. GP allows for the simultaneous optimization of economic, nutritional, and efficiency objectives, which cannot be achieved through conventional univariate statistical analysis alone.

3. Enhancing real-world applicability: By embedding real agronomic data into the GP model, the results are grounded in empirical evidence rather than theoretical assumptions. This increases the practical relevance and adoptability of the recommendations for farmers and agribusinesses.

4. Supporting evidence-based policy and farm management: The combined approach provides a transparent and reproducible method for evaluating sustainable practices under different scenarios (e.g., changing input prices, environmental regulations, or consumer preferences).

5. Methodological synergy: The experimental design ensures robust and statistically valid data, while GP translates these data into actionable insights through mathematical optimization. This synergy strengthens both the agronomic and decision-science contributions of the study.

In summary, the integration is not merely a technical exercise but a scientifically coherent approach to addressing complex, real-world problems in sustainable agriculture through a combination of empirical rigor and operational research.

Comment 2: Move regional sugarcane industry details to the Discussion or as a brief contextual note.

Response:

We thank the reviewer for this constructive suggestion regarding the flow of the manuscript. In our final revision, we have removed the detailed description of the regional sugarcane industry from the Introduction section entirely.

Upon careful reconsideration, we determined that while this context is interesting, its level of detail was not essential for framing the core research problem of optimizing mushroom cultivation substrates. Retaining it, even in a condensed form within the Discussion, could distract from the primary focus on our experimental results and their direct agronomic and economic implications.

Therefore, to maintain a sharp, concise, and focused narrative throughout the paper, we have opted to omit this specific regional industrial detail.

Comment 3: The manuscript mentions ANOVA and Tukey’s test, but key elements are missing, including Mean ± SE values in figures or tables, whether data were transformed (especially percentage and ratio data), and the Number of samples for nutritional analysis.

Response:

Thank you for this important methodological comment. We have revised the manuscript to include the requested details.

Mean ± SE values: The values in Figures 3 a, b and Tables A2 have been updated to include Mean ± Standard Error (SE). The error bars in these figures now explicitly represent the SE. Corresponding figure captions have been revised to state, Error bars represent the standard error of the mean (SE).

Data transformation: The percentage data (e.g., yield share per flush, proportion of second-grade mushrooms) and ratio data (third-flush yield ratio) were not transformed prior to ANOVA, as their residuals met the assumptions of normality and homogeneity of variance. This clarification has been added to Section 2.1: "The assumptions of normality and homogeneity of variances for ANOVA were checked and met for all variables, including percentage and ratio data; therefore, no data transformation was applied."

Sample size and preparation for nutritional analysis: The number of samples and the detailed preparation protocol for the nutritional and mineral content analysis have been clearly stated in the revised Section 2.1.10. These additions ensure full transparency and reproducibility of our statistical and analytical procedures. The relevant sections (2.1, 2.1.10, and figure captions) have been updated accordingly in the manuscript.

*** It is important to note that there is a distinction between statistical models and mathematical optimization models such as goal programming (GP). The GP model is a deterministic optimization framework, not a statistical estimation model. Its purpose is to find the optimal ranking of alternatives given a specific set of inputs (experimental data, costs, prices) and constraints. Therefore, concepts such as standard error (SE) or confidence intervals, which quantify uncertainty in estimated parameters of a statistical relationship, are not applicable to the final GP solution (the ranking itself). Uncertainty and robustness in optimization models are instead assessed through sensitivity analysis on key model inputs. In our study, this was comprehensively addressed by varying the objective weights (Table 3 and Fig. 7). This analysis shows how the optimal ranking changes with different decision-maker priorities, effectively demonstrating the model's stability and the robustness of the top-ranked alternatives to changes in these parameters. The core solution from GAMS is the single, mathematically optimal ranking for a given set of weights and data.

Comment 4: Authors are suggested to provide a full ANOVA table with all p-values and also clearly define the Main plot error and the Subplot error.

Response:

We have now provided a complete ANOVA table for the total yield data in the main text, which includes all degrees of freedom (df), sum of squares, F-values (implicitly via significance stars), and exact p-values as requested. Furthermore, we have clearly defined the error terms for the split-plot design directly in the table caption and/or the accompanying text.

Specifically:

Main Plot Error (Error a): This is the error term used to test the effect of the casing soil factor.

Subplot Error (Error b): This is the error term used to test the effect of the supplement factor and the casing soil × supplement interaction.

Additionally, in the Supplementary Materials (Appendix A2), we have provided the full set of detailed ANOVA tables for all analyzed nutritional components (protein, fiber, Cu, Mg, Mn), following the same structure with all relevant statistical parameters.

We believe these additions provide full transparency regarding the statistical analysis and meet the reviewer's request.

Comment 5: Add sample size (n) for all parameters, including mineral analysis.

Response:

Thank you for highlighting this important point. We have now explicitly stated the sample size (n) for all measured parameters throughout the Materials and Methods section (Section 2).

Specifically, for the mineral element analysis, we have added the following clarification in Section 2.1.10:

"For mineral element analysis, the sample size was n = 3. Each replicate consisted of 4 similarly-sized mushrooms, which were dried, powdered, and homogenized together to form a single composite sample per replicate."

For clarity, we also confirm that the same sample size (n = 3) was applied consistently across all other measured parameters (yield, dry matter, protein, fiber, etc.), with each replicate representing an independent experimental unit (a designated tray/m² within the randomized block design). This information is now clearly stated in the relevant methodological subsections.

Comment 6 & 7:

6. Add measures of variation (SE/SD) to Fig. 3, 5, and 6.

7. Figures lack clarity and essential information. Figures 3–6 do not include: Error bars, Sample sizes, Statistical groupings (only letters without explanation), Units on axes for nutritional index and ratios. Rectify these mistakes.

Response:

We have revised Figures 3, 5, and 6 to incorporate all requested elements:

1. Addition of Error Bars: All bar graphs and data points in the mentioned figures now include error bars representing the Standard Error of the Mean (SE). The exact SE values are also provided in the corresponding supplementary data tables.

2. Clarification of Sample Size (n): The sample size (n = 3 independent replicates per treatment) is now explicitly stated in the revised captions for Figures 3, 5, and 6. This information was also added to the Materials and Methods section (2.1.10) as per our previous response.

3. Explanation of Statistical Groupings: The figure captions now contain a clear legend explaining the meaning of the lowercase letters above the bars/data points. For example: "Different lowercase letters above bars indicate statistically significant differences between treatments according to Tukey's HSD test (p < 0.05)."

4. Axis Labels and Units:

o All axes now have complete and clear labels.

o For axes representing nutritiona

Attachment Submitted filename: response.docx

# Peer review: Decision Letter 1

**DOI:** 10.1371/journal.pone.0337146.r003

**Authors:** Nishant Kumar

20 Jan 2026

Dear Dr. Kalateh Jari,

Thank you for submitting your manuscript to PLOS ONE. After careful consideration, we feel that it has merit but does not fully meet PLOS ONE’s publication criteria as it currently stands. Therefore, we invite you to submit a revised version of the manuscript that addresses the points raised during the review process.

Please submit your revised manuscript by Mar 06 2026 11:59PM. If you will need more time than this to complete your revisions, please reply to this message or contact the journal office at plosone@plos.org. When you're ready to submit your revision, log on to https://www.editorialmanager.com/pone/ and select the 'Submissions Needing Revision' folder to locate your manuscript file.

- A letter that responds to each point raised by the academic editor and reviewer(s). You should upload this letter as a separate file labeled 'Response to Reviewers'.
- A marked-up copy of your manuscript that highlights changes made to the original version. You should upload this as a separate file labeled 'Revised Manuscript with Track Changes'.
- An unmarked version of your revised paper without tracked changes. You should upload this as a separate file labeled 'Manuscript'.

If you would like to make changes to your financial disclosure, please include your updated statement in your cover letter. Guidelines for resubmitting your figure files are available below the reviewer comments at the end of this letter.

If applicable, we recommend that you deposit your laboratory protocols in protocols.io to enhance the reproducibility of your results. Protocols.io assigns your protocol its own identifier (DOI) so that it can be cited independently in the future. For instructions see: https://journals.plos.org/plosone/s/submission-guidelines#loc-laboratory-protocols. Additionally, PLOS ONE offers an option for publishing peer-reviewed Lab Protocol articles, which describe protocols hosted on protocols.io. Read more information on sharing protocols at https://plos.org/protocols?utm_medium=editorial-email&utm_source=authorletters&utm_campaign=protocols.

We look forward to receiving your revised manuscript.

Kind regards,

Nishant Kumar, Ph.D

Academic Editor

PLOS One

**Journal Requirements:**

1. If the reviewer comments include a recommendation to cite specific previously published works, please review and evaluate these publications to determine whether they are relevant and should be cited. There is no requirement to cite these works unless the editor has indicated otherwise.

[Note: HTML markup is below. Please do not edit.]

Reviewers' comments:

Reviewer's Responses to Questions

**Comments to the Author**

Reviewer #1: All comments have been addressed

Reviewer #2: All comments have been addressed

**********

2. Is the manuscript technically sound, and do the data support the conclusions?

Reviewer #1: Yes

Reviewer #2: Yes

**********

3. Has the statistical analysis been performed appropriately and rigorously?

Reviewer #1: Yes

Reviewer #2: No

**********

4. Have the authors made all data underlying the findings in their manuscript fully available?

The PLOS Data policy

Reviewer #1: Yes

Reviewer #2: Yes

**********

5. Is the manuscript presented in an intelligible fashion and written in standard English?

Reviewer #1: Yes

Reviewer #2: Yes

**********

Reviewer #1: (No Response)

Reviewer #2: The authors have addressed most of the reviewers comments. In my opinion, the manuscript still reads more like an applied agronomic study than a mechanistically grounded contribution to food/biochemical or bioprocess science, which may remain a concern for editorial acceptance.

1. The abstract now mentions “metabolic and enzymatic pathways”, but no specific enzymes, transporters or biosynthetic routes were included. The claims remain largely conceptual and not supported by experimental validation.

2. No dose–response framework or physiological threshold discussion is included. Specify why specific mineral precursors were selected or whether they act as enzyme cofactors, transcriptional regulators, or structural elements

3. Increase in Fe/Zn/Mg is reported but still no discussion on, competitive ion uptake, transport saturation or potential antagonistic effects between minerals.

4. Tables again lack sample size (n).

5. Figures do not include, error bar definition (SD vs. SE)

6. Ethical, safety, and regulatory context still missing, Food safety thresholds for mineral fortification, regulatory limits (FSSAI/WHO/EFSA), add discussion on these aspects.

In my opinion, the MS still requires one more round of review.

**********

what does this mean?). If published, this will include your full peer review and any attached files.

If you choose “no”, your identity will remain anonymous but your review may still be made public.

**Do you want your identity to be public for this peer review?**  For information about this choice, including consent withdrawal, please see our Privacy Policy

Reviewer #1: No

Reviewer #2: **Yes:**  Aditya, Ph.D.

**********

[NOTE: If reviewer comments were submitted as an attachment file, they will be attached to this email and accessible via the submission site. Please log into your account, locate the manuscript record, and check for the action link "View Attachments". If this link does not appear, there are no attachment files.]

To ensure your figures meet our technical requirements, please review our figure guidelines: https://journals.plos.org/plosone/s/figures

You may also use PLOS’s free figure tool, NAAS, to help you prepare publication quality figures: https://journals.plos.org/plosone/s/figures#loc-tools-for-figure-preparation.

NAAS will assess whether your figures meet our technical requirements by comparing each figure against our figure specifications.

# Author comment: Author response to Decision Letter 2

**DOI:** 10.1371/journal.pone.0337146.r004

1 May 2026

Dear Editors and Reviewers,

We hope this message finds you well. We are pleased to submit the revised version of our manuscript entitled “Economic, Nutritional, and Efficiency Assessment of Recycled Casing Soil and Compost Nutritional Supplements for Sustainable White Button Mushroom Cultivation.”

We have carefully considered all the reviewers' comments and have revised the manuscript accordingly. For clarity, each reviewer's comments have been addressed in the following color-coded sections:

• Reviewer 2: Pink highlights

• Reviewer 3: Green highlights

We believe that the revisions have significantly strengthened the manuscript, and we are grateful for the insightful and constructive feedback provided by the reviewers.

Thank you for your time and consideration.

Reviewer #2:

The authors have addressed most of the reviewers comments. In my opinion, the manuscript still reads more like an applied agronomic study than a mechanistically grounded contribution to food/biochemical or bioprocess science, which may remain a concern for editorial acceptance.

Comment 1: The abstract now mentions “metabolic and enzymatic pathways”, but no specific enzymes, transporters or biosynthetic routes were included. The claims remain largely conceptual and not supported by experimental validation.

Response: We truly appreciate the reviewer’s time and careful reading of our manuscript. That said, we’d like to gently point out that the phrase “metabolic and enzymatic pathways” never actually appears in the abstract of any version we submitted. Our abstract—and really the whole manuscript—focuses on economic profitability, nutritional quality, and production efficiency, all within a goal‑programming framework for sustainable mushroom cultivation. The main contribution of our work is a multi‑objective model that combines experimental agronomic data with economic and nutritional indicators, not a mechanistic deep dive into molecular pathways.

Still, to address the reviewer’s concern about mechanistic depth, we’ve added brief discussions of relevant enzymes (like superoxide dismutase, ATPases, and tyrosinase) and transporters (such as the ZIP family, MgtE, and NRAMP) in both the Introduction and Discussion sections—these changes are detailed in our responses to comments #2 and #3. We hope these clarifications and additions strike the right balance and satisfy the reviewer’s concern, while keeping the focus where it belongs: on the applied agronomic and economic nature of our study.

Comment 2: No dose–response framework or physiological threshold discussion is included. Specify why specific mineral precursors were selected or whether they act as enzyme cofactors, transcriptional regulators, or structural elements.

Response: We appreciate the reviewer raising this important point. To address it, we’ve made substantial revisions to the manuscript, adding the requested dose–response framework, physiological thresholds, and a clear rationale for selecting Mg, Zn, Cu, and Mn. Here’s a summary of the key changes.

In the Introduction (new paragraph after line 5), we now explicitly explain why we chose these four minerals. Specifically, we discuss their well‑established physiological roles in Agaricus bisporus—as enzyme cofactors (e.g., Zn in superoxide dismutase, Mg in ATPases, Cu in cytochrome c oxidase, Mn in Mn‑SOD), as regulators (Zn‑finger proteins), and as structural components (Mg in ribosomes). This directly responds to the reviewer’s request to clarify the mode of action of these mineral precursors.

In the Results (section 3.2.2, Nutritional characteristics analysis), we’ve added the dose‑response analysis. We now describe that increasing soybean meal from 1000 to 1500 g m⁻² led to a substantial rise in the Food Elements Index (FEI), reflecting a clear linear dose‑dependent pattern. In contrast, corn grit showed only a marginal increase beyond 1000 g m⁻², suggesting transport saturation already at the lower dose. These observations are presented without over‑specifying numerical values beyond our experimental resolution.

In the Discussion, we inserted a new mechanistic paragraph right after the sentence beginning “The observed inverse relationship between high yield and superior nutritional values…”. There we explain the physiological thresholds for nitrogen (soybean meal) and carbohydrate (corn grit) supplementation. For soybean meal, we note that 1500 g m⁻² approaches the nitrogen tolerance threshold; above that, ammonia‑mediated stress would likely compromise yield. For corn grit, the near‑saturation of mineral uptake at 1000 g m⁻² points to a finite transport capacity for divalent cations, probably mediated by ZIP‑family transporters. This paragraph also revisits the cofactor and structural roles of the selected minerals, linking the dose‑response patterns directly to metabolic saturation.

Together, these revisions provide the previously missing dose‑response framework, clarify the physiological basis for our mineral selection, and address all three mechanistic categories requested by the reviewer (enzyme cofactors, transcriptional regulators, and structural elements). We believe the manuscript is now much stronger in this respect.

Comment 3: increase in Fe/Zn/Mg is reported but still no discussion on, competitive ion uptake, transport saturation or potential antagonistic effects between minerals.

Response: We’ve gone back and substantially revised the Discussion to better address competitive uptake, transport saturation, and antagonistic effects—specifically for the cations we actually measured: Mg, Zn, Cu, and Mn. The reviewer mentioned iron, but to be clear, Fe wasn't part of our mineral panel. Our rationale, which we've already laid out in the Introduction, focuses on Mg, Zn, Cu, and Mn because of their established roles in human health. That said, we didn't ignore the mechanistic side. The revised Discussion now covers three key points: (i) the shared ZIP‑family transporters for Zn²⁺ and Cu²⁺ and the resulting potential for competition; (ii) clear evidence of transport saturation, as seen in the plateau effect with corn grit; and (iii) antagonistic interactions—specifically between Ca²⁺ (from the CaCO₃ in the casing layer) and Mg²⁺, as well as between Zn²⁺ and Cu²⁺.

Comment 4: Tables again lack sample size (n).

Response: We thank the reviewer for this careful observation.

In Table 3 (ANOVA table), the degrees of freedom (total df = 104, error df = 56) reflect the underlying sample size of 105 experimental units (35 treatments × 3 replications). However, to make this explicit for readers, we have added a footnote to Table 3 stating: "The total number of experimental units was 105 (35 treatments × 3 replicates)."

We have now added an explicit statement of sample size to all relevant tables and figures. Specifically, we added the following note to Tables 3, 4, and Appendix A3, as well as to Figures 3, 4, 5, and 6:

"Note: Each treatment combination was replicated three times (n = 3 per treatment)"

This note now appears alongside each table and figure where experimental data are presented. We believe this addresses the reviewer's concern completely. Additionally, the sample size (n = 3) was already explicitly stated in the Materials and Methods section (sections 2.1.2 and 2.1.10).

Thank you again for the valuable feedback.

Comment 5: Figures do not include, error bar definition (SD vs. SE)

Response: We thank the reviewer for this observation. We have added error bars to all figures (Figures 3, 4, 5, and 6) and have clearly defined the type of error bar used.

Error bars represent Standard Error of the Mean (SE), not Standard Deviation (SD). We chose SE because it is the appropriate measure for showing the precision of the sample mean when comparing treatment effects.

A note stating "Error bars represent the standard error of the mean (±SE) calculated from the raw data" has been added beneath each figure (Figures 3, 4, 5, and 6).

The definition of SE (SE = SD / √n) and the sample size (n = 3) are explicitly stated in the Materials and Methods section (sections 2.1.2 and 2.1.10).

Comment 6: Ethical, safety, and regulatory context still missing, Food safety thresholds for mineral fortification, regulatory limits (FSSAI/WHO/EFSA), add discussion on these aspects.

Response: Thanks to comment #6, we've now added a dedicated paragraph in the Discussion—right before the Conclusion—that directly addresses food safety. Specifically, we reference the relevant guidelines from EFSA, WHO/FAO, and FSSAI for Zn, Cu, and Mg, convert our dry‑weight concentrations into estimated intake per serving on a fresh‑weight basis, and confirm that all values stay within the tolerable upper intake levels. We also note that there are currently no regulatory limits specifically for these minerals in edible mushrooms. The paragraph includes a brief ethical note (no approval required) and a practical recommendation: periodic heavy metal monitoring when recycled casing soil is being reused. This addition fills the regulatory and safety gap the reviewer pointed out.

Reviewer #3:

Comment 1: The introduction is unusually long, overly descriptive, and contains repeated justifications.

Response: We thank the reviewer for this observation. We have thoroughly revised the Introduction to make it more concise while preserving all original references and the core logical structure. The revised version is approximately 30-35% shorter. Specifically, we:

• Removed redundant justifications (e.g., repeated explanations of mushroom benefits and peat extraction problems that appeared in multiple paragraphs).

• Condensed descriptive passages without removing any citations.

• Streamlined the paragraph on goal programming by eliminating repetitive explanations of its philosophy.

• Restructured the novelty paragraph to be more direct and less verbose.

The revised Introduction is now more focused and reader-friendly while retaining all original references and technical content.

Comment 2: Why not use multi-criteria methods like: TOPSIS, AHP, MOORA, Multi-objective linear programming directly (without ranking)?

Response: We thank the reviewer for this insightful methodological question. The choice of goal programming (GP) over other multi-criteria methods was deliberate and is justified by the specific nature of our problem, as explained below.

1. The nature of our objectives: targets, not just rankings

Methods such as TOPSIS, AHP, and MOORA are primarily designed to rank alternatives based on their relative closeness to an ideal solution or pairwise comparisons. However, our decision problem involves three conflicting objectives for which we have specific target values derived from experimental data. Goal programming is uniquely suited for such problems because it minimizes deviations from predefined targets (whether shortfall or excess), rather than merely producing a relative ranking. This target-based approach aligns better with real-world decision-making, where growers have aspirational goals (e.g., achieving a certain profit level or limiting third-flush yield to below 10%) rather than simply seeking the "best" among imperfect options.

2. The discrete nature of our alternatives

Multi-objective linear programming (direct optimization without ranking) typically assumes continuous decision variables (e.g., how much of each ingredient to mix). In our study, however, the alternatives are discrete – they arise from specific, predefined combinations of casing formulations (C1 to C7) and supplement levels (S1 to S5) tested in the field experiment. These 35 discrete alternatives cannot be represented as continuous variables. Therefore, a discrete multi-criteria decision-making (MCDM) approach, such as GP with binary variables, is methodologically appropriate, whereas continuous multi-objective LP is not feasible for this problem structure.

3. Robustness through sensitivity analysis

While AHP is powerful for deriving weights from pairwise comparisons, it assumes that the decision-maker can consistently compare all pairs of criteria – a demanding task when three objectives are incommensurable. Our GP model, in contrast, allows direct specification of weights (based on producer priorities) and includes a comprehensive Monte Carlo simulation (100 random weight vectors per scenario) to test the robustness of rankings. This level of sensitivity analysis is less common in standard TOPSIS or MOORA applications.

4. Alignment with real-world producer priorities

In practice, mushroom growers do not think in terms of "which alternative is closest to an ideal point." Instead, they ask: "Given my profit target, my desired nutritional quality, and my willingness to accept a longer growing cycle, which combination minimizes my disappointment across all goals?" This is precisely what GP does: it minimizes the weighted sum of deviations from targets. The ability to assign different weights to positive and negative deviations (over-achievement vs. under-achievement) is another strength of GP not shared by TOPSIS or MOORA.

For the reasons above – target-based deviation minimization, discrete alternative structure, robustness via sensitivity analysis, and alignment with real-world decision logic – goal programming was the most appropriate method for our study. We thank the reviewer again for the opportunity to clarify this important point.

Comment 3: The figures need Improvement as many graphs lack error bars and Axis labels are not visible or too small.

Response: We thank the reviewer for this observation. Error bars have been added to all figures (Figures 3, 4, 5, and 6). A clear definition has been provided beneath each figure stating: "Error bars represent the standard error of the mean (±SE) calculated from the raw data." We used SE (not SD) as it is the appropriate measure for the precision of the sample mean. We have also increased the font size of all axis labels to ensure they are clearly visible. All figures have been regenerated with higher resolution. The revised figures now meet the journal's standards.

Comment 4: The result section reads like a raw extraction from analyses. More synthesis is needed.

Response: The reviewer is correct. We have substantially rewritten the Results section to move beyond mere description of numbers and to provide meaningful synthesis. Specifically, we made the following revisions:

• We began each subsection with a topic sentence that states the main pattern or finding.

• We added interpretive statements that explain why a pattern matters (for example, we explained that the 47% yield difference between supplements indicates supplement choice is more critical than casing type).

• We explicitly highlighted trade-offs, such as the inverse relationship between high-yield and high-nutrition formulations.

• We drew practical implications where appropriate, including that corn grit at 1500 g/m² allows early termination of the third flush with associated energy and cost savings.

All original data, numbers, statistical results, and references remain unchanged. Only the presentation has been enhanced to provide synthesis and interpretation. These revisions have been completed throughout section 3.

Comment 5: Several sentences are very long, contain redundancies, or use overly formal/unnecessary phrasing.

Response: We have carefully edited the entire manuscript to improve sentence fluency. Specific changes include:

Breaking overly long sentences into shorter, clearer ones.

Removing redundant phrases such as "in other words," "thereby," "constitutes the only," and "concurrently."

Replacing overly formal constructions (e.g., "can be attributed to" → "stems from"; "the findings clearly demonstrate" → no unnecessary adverb).

Eliminating unnecessary adjectives and adverbs without changing meaning.

Comment 6: Rewrite your Abstract

Response: The Abstract has been completely rewritten. The revised version is more concise, result-oriented, and follows a clear structure: problem statement, methods, key results, and conclusion. Unnecessary descriptive phrases have been removed, and the main quantitative finding (optimal combination C5+S5) is now stated upfront.

Comment 7: Improve the discussion

Response: The Discussion has been substantially imp

Attachment Submitted filename: Response to Reviewers_Round 2.docx

# Peer review: Decision Letter 2

**DOI:** 10.1371/journal.pone.0337146.r005

**Authors:** Nishant Kumar

9 Jun 2026

Dear Dr. Jari,

Thank you for submitting your manuscript to PLOS ONE. After careful consideration, we feel that it has merit but does not fully meet PLOS ONE’s publication criteria as it currently stands. Therefore, we invite you to submit a revised version of the manuscript that addresses the points raised during the review process.

==============================

**ACADEMIC EDITOR: N/A**

==============================

Please submit your revised manuscript by Jul 24 2026 11:59PM. If you will need more time than this to complete your revisions, please reply to this message or contact the journal office at plosone@plos.org. When you're ready to submit your revision, log on to https://www.editorialmanager.com/pone/ and select the 'Submissions Needing Revision' folder to locate your manuscript file.

- A letter that responds to each point raised by the academic editor and reviewer(s). You should upload this letter as a separate file labeled 'Response to Reviewers'.
- A marked-up copy of your manuscript that highlights changes made to the original version. You should upload this as a separate file labeled 'Revised Manuscript with Track Changes'.
- An unmarked version of your revised paper without tracked changes. You should upload this as a separate file labeled 'Manuscript'.

If you would like to make changes to your financial disclosure, please include your updated statement in your cover letter. Guidelines for resubmitting your figure files are available below the reviewer comments at the end of this letter.

If applicable, we recommend that you deposit your laboratory protocols in protocols.io to enhance the reproducibility of your results. Protocols.io assigns your protocol its own identifier (DOI) so that it can be cited independently in the future. For instructions see: https://journals.plos.org/plosone/s/submission-guidelines#loc-laboratory-protocols. Additionally, PLOS ONE offers an option for publishing peer-reviewed Lab Protocol articles, which describe protocols hosted on protocols.io. Read more information on sharing protocols at https://plos.org/protocols?utm_medium=editorial-email&utm_source=authorletters&utm_campaign=protocols.

As the corresponding author, your ORCID iD is verified in the submission system and will appear in the published article. PLOS supports the use of ORCID, and we encourage all coauthors to register for an ORCID iD and use it as well. Please encourage your coauthors to verify their ORCID iD within the submission system before final acceptance, as unverified ORCID iDs will not appear in the published article. *Only*  the individual author can complete the verification step; PLOS staff *cannot*  verify ORCID iDs on behalf of authors.

We look forward to receiving your revised manuscript.

Kind regards,

Nishant Kumar, Ph.D

Academic Editor

PLOS One

Journal Requirements:

If the reviewer comments include a recommendation to cite specific previously published works, please review and evaluate these publications to determine whether they are relevant and should be cited. There is no requirement to cite these works unless the editor has indicated otherwise.

Please review your reference list to ensure that it is complete and correct. If you have cited papers that have been retracted, please include the rationale for doing so in the manuscript text, or remove these references and replace them with relevant current references. Any changes to the reference list should be mentioned in the rebuttal letter that accompanies your revised manuscript. If you need to cite a retracted article, indicate the article’s retracted status in the References list and also include a citation and full reference for the retraction notice.

[Note: HTML markup is below. Please do not edit.]

Reviewers' comments:

Reviewer's Responses to Questions

**Comments to the Author**

Reviewer #2: All comments have been addressed

**********

2. Is the manuscript technically sound, and do the data support the conclusions?

Reviewer #2: Yes

**********

3. Has the statistical analysis been performed appropriately and rigorously?

Reviewer #2: Yes

**********

4. Have the authors made all data underlying the findings in their manuscript fully available?

The PLOS Data policy

Reviewer #2: Yes

**********

5. Is the manuscript presented in an intelligible fashion and written in standard English?

Reviewer #2: Yes

**********

Reviewer #2: The revised manuscript has addressed most of the major reviewer concerns satisfactorily. However, few minor revisions are still required before moving ahead.

1. In the discussion section (Pages 26–30), several explanations regarding transporter activity, enzymatic regulation and nutrient uptake mechanisms are presented too definitively despite the absence of molecular, enzymatic or metabolomic validation. These statements should be revised as probable physiological explanations rather than experimentally confirmed mechanisms.

2. Although regulatory discussion has been added by the authors, but I still recommend to add possible microbial contamination risks, heavy metal accumulation concerns associated with recycled casing soil. A brief limitation statement should be included in the discussion section.

3. The authors can avoid using terms such as “innovative framework,” “methodological breakthrough,” and similar promotional terms.

4. Some sections, particularly the Goal Programming methodology and Discussion, remain repetitive. Avoid repetitions.

5. Check all in-text citations with full references.

**********

what does this mean?). If published, this will include your full peer review and any attached files.

If you choose “no”, your identity will remain anonymous but your review may still be made public.

**Do you want your identity to be public for this peer review?**  For information about this choice, including consent withdrawal, please see our Privacy Policy

Reviewer #2: **Yes:**  Dr. Aditya Bhatia

**********

[NOTE: If reviewer comments were submitted as an attachment file, they will be attached to this email and accessible via the submission site. Please log into your account, locate the manuscript record, and check for the action link "View Attachments". If this link does not appear, there are no attachment files.]

To ensure your figures meet our technical requirements, please review our figure guidelines: https://journals.plos.org/plosone/s/figures

You may also use PLOS’s free figure tool, NAAS, to help you prepare publication quality figures: https://journals.plos.org/plosone/s/figures#loc-tools-for-figure-preparation.

NAAS will assess whether your figures meet our technical requirements by comparing each figure against our figure specifications.

# Author comment: Author response to Decision Letter 3

**DOI:** 10.1371/journal.pone.0337146.r006

25 Jun 2026

Response to Reviewers

Manuscript ID: PONE-D-25-59546R2

Title: Economic, Nutritional, and Efficiency Assessment of Recycled Casing Soil and Compost Nutritional Supplements for Sustainable White Button Mushroom Cultivation

Journal: PLOS ONE

Dear Editor and Reviewer 2,

We sincerely thank the reviewer for the constructive comments on our revised manuscript. We are pleased that the major concerns have been addressed satisfactorily. Below we respond to each remaining minor revision point by point.

Response to Reviewer 2

Comment 1:

"In the discussion section (Pages 26–30), several explanations regarding transporter activity, enzymatic regulation and nutrient uptake mechanisms are presented too definitively despite the absence of molecular, enzymatic or metabolomic validation. These statements should be revised as probable physiological explanations rather than experimentally confirmed mechanisms."

Response: We agree and have carefully revised the Discussion section accordingly. All mechanistic interpretations—including transporter activity, ion antagonism, and ammonia‑induced toxicity—are now presented as plausible physiological explanations or testable hypotheses rather than confirmed mechanisms. Definitive language such as "reflects" has been replaced with more cautious phrasing such as "may be partially explained by", "is consistent with", and "suggests". We have also explicitly stated that these interpretations require direct molecular, enzymatic, or transcriptomic validation in future studies.

Comment 2:

"Although regulatory discussion has been added by the authors, but I still recommend to add possible microbial contamination risks, heavy metal accumulation concerns associated with recycled casing soil. A brief limitation statement should be included in the discussion section."

Response: We have added a dedicated limitation paragraph in the Discussion section that explicitly addresses: (i) potential microbial contamination risks (e.g., Pseudomonas spp., Trichoderma spp.) with repeated reuse of recycled casing soil; (ii) possible gradual accumulation of non‑essential heavy metals (e.g., Cd, Pb) over multiple cycles; (iii) the single‑cycle scope of our data; and (iv) the hypothetical nature of our proposed physiological mechanisms. This paragraph has been inserted following the circular economy benefits discussion.

Comment 3:

"The authors can avoid using terms such as 'innovative framework,' 'methodological breakthrough,' and similar promotional terms."

Response: We have removed all promotional and overly assertive language throughout the manuscript. For example, "Our primary innovation is" has been changed to "This study presents"; "methodological breakthrough" has been replaced with "approach"; and "novel decision‑making framework" has been revised to "decision‑making framework". Similar neutralizations have been applied consistently across the Abstract, Introduction, and Conclusion.

Comment 4:

"Some sections, particularly the Goal Programming methodology and Discussion, remain repetitive. Avoid repetitions."

Response: We have eliminated redundancies as follows. First, the detailed GP explanation in the Introduction has been condensed to a brief 2‑line summary that refers readers to Section 2.2 for the full formulation. Second, numerical yield values (e.g., 36,669 g/m²) have been removed from the Discussion and replaced with references to Section 3.1 and Fig. 3c. Third, the repeated comparison between C1S4 and C1S3 in the Discussion has been streamlined with a concise reference to Section 3.2.2 and Fig. 5.

Comment 5:

"Check all in‑text citations with full references."

Response: We have meticulously cross‑checked every in‑text citation against the reference list. All newly added references are correctly numbered, and no retracted papers were identified. All citations now appear in the correct order and format according to PLOS ONE guidelines.

Summary of Revisions

All changes described above are clearly highlighted in the "Revised Manuscript with Track Changes" file. An unmarked clean version of the manuscript is also provided. In summary: mechanistic interpretations in the Discussion have been softened to hypothetical explanations; a new limitation paragraph addresses microbial and heavy metal risks; promotional language has been removed; redundancies between sections have been eliminated; and all in‑text citations have been verified against the reference list.

We believe the revised manuscript now fully addresses all remaining concerns and hope it is acceptable for publication in PLOS ONE.

Yours sincerely,

Corresponding Author

Attachment Submitted filename: Response to Reviewers.docx

# Editor report: Decision Letter 3

**DOI:** 10.1371/journal.pone.0337146.r007

**Authors:** Nishant Kumar

28 Jun 2026

Economic, Nutritional, and Efficiency Assessment of Recycled Casing Soil and Compost Nutritional Supplements for Sustainable White Button Mushroom Cultivation

PONE-D-25-59546R3

Dear Dr. Jari,

We’re pleased to inform you that your manuscript has been judged scientifically suitable for publication and will be formally accepted for publication once it meets all outstanding technical requirements.

Within one week, you’ll receive an e-mail detailing the required amendments. When these have been addressed, you’ll receive a formal acceptance letter and your manuscript will be scheduled for publication.

An invoice will be generated when your article is formally accepted. Please note, if your institution has a publishing partnership with PLOS and your article meets the relevant criteria, all or part of your publication costs will be covered. Please make sure your user information is up-to-date by logging into Editorial Manager at Editorial Manager® and clicking the ‘Update My Information' link at the top of the page. For questions related to billing, please contact billing support.

If your institution or institutions have a press office, please notify them about your upcoming paper to help maximize its impact. If they’ll be preparing press materials, please inform our press team as soon as possible -- no later than 48 hours after receiving the formal acceptance. Your manuscript will remain under strict press embargo until 2 pm Eastern Time on the date of publication. For more information, please contact onepress@plos.org.

Kind regards,

Nishant Kumar, Ph.D

Academic Editor

PLOS One

Additional Editor Comments (optional):

Reviewers' comments:

# Editor report: Acceptance letter

**DOI:** 10.1371/journal.pone.0337146.r008

**Authors:** Nishant Kumar

PONE-D-25-59546R3

PLOS One

Dear Dr. Kalateh Jari,

I'm pleased to inform you that your manuscript has been deemed suitable for publication in PLOS One. Congratulations! Your manuscript is now being handed over to our production team.

At this stage, our production department will prepare your paper for publication. This includes ensuring the following:

* All references, tables, and figures are properly cited

* All relevant supporting information is included in the manuscript submission,

* There are no issues that prevent the paper from being properly typeset

You will receive further instructions from the production team, including instructions on how to review your proof when it is ready. Please keep in mind that we are working through a large volume of accepted articles, so please give us a few days to review your paper and let you know the next and final steps.

Lastly, if your institution or institutions have a press office, please let them know about your upcoming paper now to help maximize its impact. If they'll be preparing press materials, please inform our press team within the next 48 hours. Your manuscript will remain under strict press embargo until 2 pm Eastern Time on the date of publication. For more information, please contact onepress@plos.org.

You will receive an invoice from PLOS for your publication fee after your manuscript has reached the completed accept phase. If you receive an email requesting payment before acceptance or for any other service, this may be a phishing scheme. Learn how to identify phishing emails and protect your accounts at https://explore.plos.org/phishing.

If we can help with anything else, please email us at customercare@plos.org.

Thank you for submitting your work to PLOS ONE and supporting open access.

Kind regards,

PLOS ONE Editorial Office Staff

on behalf of

Dr. Nishant Kumar

Academic Editor

PLOS One
